# Genetic Dissection of Nitrogen Use Efficiency in Tropical Maize Through Genome-Wide Association and Genomic Prediction

**DOI:** 10.3389/fpls.2020.00474

**Published:** 2020-04-28

**Authors:** Berhanu Tadesse Ertiro, Maryke Labuschagne, Michael Olsen, Biswanath Das, Boddupalli M. Prasanna, Manje Gowda

**Affiliations:** ^1^Bako National Maize Research Center, Ethiopian Institute of Agricultural Research, Bako, Ethiopia; ^2^International Maize and Wheat Improvement Center, World Agroforestry Centre, Nairobi, Kenya; ^3^Department of Plant Sciences, University of the Free State, Bloemfontein, South Africa

**Keywords:** association mapping, NUE, SNP, marker-assisted selection, LD, genomic prediction

## Abstract

In sub-Saharan Africa, one of the major challenges to smallholder farmers is soil with low fertility and inability to apply nitrogen fertilizer externally due to the cost. Development of maize hybrids, which perform better in nitrogen depleted soils, is one of the promising solutions. However, breeding maize for nitrogen use efficiency (NUE) is hindered by expensive phenotypic evaluations and trait complexity under low N stress. Genome-wide association study (GWAS) and genomic prediction (GP) are promising tools to circumvent this interference. Here, we evaluated a mapping panel in diverse environments both under optimum and low N management. The objective of this study was to identify SNPs significantly associated with grain yield (GY) and other traits through GWAS and assess the potential of GP under low N and optimum conditions. Testcross progenies of 411 inbred lines were planted under optimum and low N conditions in several locations in Africa and Latin America. In all locations, low N fields were previously depleted over several seasons, and no N fertilizer was applied throughout the growing season. All inbred lines were genotyped with genotyping by sequencing. Genotypic and GxE interaction variances were significant, and heritability estimates were moderate to high for all traits under both optimum and low N conditions. Genome-wide LD decay at *r*^2^ = 0.2 and *r*^2^ = 0.34 were 0.24 and 0.19 Mbp, respectively. Chromosome-specific LD decays ranged from 0.13 to 0.34 Mbps with an average of 0.22 Mbp at *r*^2^ = 0.2. GWAS analyses revealed 38 and 45 significant SNPs under optimum and low N conditions, respectively. Out of these 83 significant SNPs, 3 SNPs on chromosomes 1, 2, and 6 were associated either with different traits or the same trait under different management conditions, suggesting pleiotropic effects of genes. A total of 136 putative candidate genes were associated with the significant SNPs, of which seven SNPs were linked with four known genes. Prediction accuracies were moderate to high for all traits under both optimum and low N conditions. These results can be used as useful resources for further applications to develop hybrids or lines with better performance under low N conditions.

## Introduction

Low N stress is a widespread problem for maize production in sub-Saharan Africa (SSA) particularly in smallholder farmers. The average fertilizer use in SSA was around 8 kg ha^–1^, which is far below compared to other regions in the developing world (Morris et al., 2007; Das et al., 2019). Even though in 2006, all members of the African Union pledged to increase the level of fertilizer use substantially by 2015 (African Development Bank, 2006), till now, the fertilizer use in SSA remains less than 10 kg ha^–1^ (FAOSTAT, 2017). Fertilizers in SSA are very expensive, and smallholder farmers can hardly afford the application of right amount and kind of fertilizer required for the normal growth and development of maize (Lafitte and Edmeades, 1994; Das et al., 2019). In SSA, smallholder farmers grow maize with less or no fertilizer on already severely N depleted soils; as a result, yield decreases drastically to <2 tons per hectare (t/ha). Nevertheless, developing maize varieties that can perform better under low N conditions can offer better intervention to increase yields in the field of smallholder farmers’ (Cairns et al., 2012). Currently very few breeding programs are targeting on developing low-N tolerance varieties (Das et al., 2019). Classical breeding based correlation studies point to distinct genetic mechanisms for grain yield (GY) under optimum and under low N stressed conditions (Bänziger et al., 1997; Worku et al., 2007, 2008). Further, most of economically important traits are controlled by multiple quantitative trait loci (QTLs) and are difficult to investigate only with conventional approaches *per se*.

Molecular markers brought most significant developments in the field of genetics and plant breeding (Soto-Cerda and Cloutier, 2010). Specifically genetic mapping and linking markers to candidate genes or functional loci facilitated genome-aided breeding for crop improvements including nitrogen use efficiency (NUE) and drought tolerance (Yu and Buckler, 2006). Linkage analysis has been widely used in dissecting the genetic basis of economically important complex traits in plants. Several such studies have been conducted to understand the genetic architecture of GY and other agronomic traits under different environmental conditions (Ribaut et al., 2007). Despite many studies on linkage analysis conducted in various crop plants to dissect the quantitative traits, only a few QTLs were cloned or tagged at the gene level (Moose and Mumm, 2008) as cloning of QTLs is time consuming and laborious (Yan et al., 2011). Since only two alleles per locus and a few recombination events are considered to identify the QTL, this leads to limited mapping resolution (Soto-Cerda and Cloutier, 2010).

Genome-wide association mapping can be able to capture complex trait variation down to the sequence level by exploiting both historical as well as evolutionary recombination events (Nordborg and Tavare, 2002). This approach was initially started in human disease studies and then was extended to plants, substantially increasing the mapping resolution over the traditional linkage mapping (Flint-Garcia et al., 2003). Association mapping detects the correlation between genotype and phenotype in unrelated individuals based on linkage disequilibrium (LD). It identifies QTL by probing the marker–trait associations, which is attributed to the strength of LD between markers and functional polymorphisms in diverse germplasm (Zhu et al., 2008). With association studies, mapping resolution increased, and reduced in time to develop population/s, and accounts for a greater allele number (Flint-Garcia et al., 2003; Yu and Buckler, 2006) compared to the traditional linkage analysis.

A number of association mapping studies have been conducted to investigate the causal variants linked with many important traits in maize, including flowering time (Wallace et al., 2016), forage quality (Anderson et al., 2007), carotenoid content (Harjes et al., 2008), provitamin A (Azmach et al., 2013), and kernel size (Li et al., 2010). Despite the widespread use of association mapping for the dissection of complex traits, little was done for the dissection of the genetic architecture of NUE in maize. NUE is a complex trait that is a product of N uptake and N utilization efficiency. In addition to limited work on NUE, genome-wide association studies (GWASs) are constrained by the power of statistical tools to identity true associations, calling for better computer software packages for data analysis.

The application of GWAS has been limited by the presence of false positives and false negatives. Significant results from different association studies have hardly been reproducible due to false positives resulting from population structure, which is a major problem for association mapping (Zhu et al., 2008). In general, association panel has a set of lines with different geographical origins, local adaptation, and breeding history; as a result, these lines usually contain both population structure and familial relatedness (Yu and Buckler, 2006). In order to avoid spurious results, LD generated by the population structure within the panel has to be considered in the analysis. In a mixed linear model (MLM), both the population structure and kinship among individuals are incorporated as covariates to control the false positives. However, the confounding effect between the covariates and the test marker weakens the signals of QTNs (quantitative trait nucleotides); as a result, false negatives increase (Liu et al., 2016). Recently, a user-friendly R GWAS package known as FarmCPU (Fixed and random model Circulating Probability Unification) implemented a method to address the “confounding effect” and increase the speed and save memory by using several programming strategies, which makes FarmCPU to be adapted for big data sets (Liu et al., 2016). In this study “the state-of-the-art” analytical package was used to identify the marker–trait association in testcrosses of 411 tropical inbred lines evaluated under optimum and low N conditions.

Genomic selection (GS) is another potential tool that uses uniformly distributed dense molecular markers across the genome to predict the performance of individuals of known genotype but unknown phenotype (Zhang et al., 2015). GS has been widely used in maize breeding, on GY, and other traits (Zhang et al., 2015, 2017; Beyene et al., 2019; Yuan et al., 2019), which clearly demonstrated its potential in improving quantitative traits. GWAS empowers the detection of QTNs for the target trait by using a diverse set of breeding lines, whereas GS enables the selection of superior individuals by considering the effects of multiple genes controlling a target trait (Crossa et al., 2017; Yuan et al., 2019). Combining GWAS and GS with marker assisted selection (MAS) accelerates the breeding efficiency to develop the lines or hybrids with better performance for GY and other complex traits under diverse management conditions. Therefore, the full potential of these two tools needs to be assessed using a set of elite lines and/or practical breeding populations for NUE.

The objectives of the study were to (1) evaluate the diverse set of tropical and subtropical maize lines for their responses to GY and other yield-related traits under optimum and low N stress conditions; (2) identify the genomic regions, QTNs, and putative candidate genes associated with these traits under both management conditions; and (3) assess the potential of GS within and across management conditions. This study will provide useful information for uncovering the genetic basis of NUE and design the MAS scheme for breeding high NUE maize.

## Materials and Methods

### Plant Material

Four hundred and eleven inbred lines used for this study were derived from a panel of 424 diverse tropical maize inbred lines established by the Improved Maize for African Soils (IMAS) project to dissect the genetic basis of NUE and for marker discovery. All the inbred lines were CIMMYT maize lines developed by CIMMYT through conventional breeding methods. The list of the inbred lines, the source germplasm, and the method employed for the development of the lines can be found at http://www.data.cimmyt.org. Single cross hybrids were generated for the evaluation of the inbred lines by crossing with CML539, a broadly adapted CIMMYT maize inbred line tester from heterotic group A.

### Field Experiments and Statistical Analysis

Testcross progenies were evaluated across 9 optimum and 13 managed low N stressed sites in Africa and Latin America. The list of the trials, testing sites, and the management practices employed for each trial are presented in Table 1. For managed low N sites, the soil was depleted up to 6 years by planting sorghum in high density. An alpha-lattice design was used, with two replications. Experiments were planted in one-row plots, with a final planting density of 6.67 plants/m^2^ (Mexico) and 5.33 plants/m^2^ (Kenya, South Africa, Zambia, and Zimbabwe). At all locations, two seeds per hill were sown, and then thinned to one after emergence. For optimal trials, the recommended amount of fertilizer was applied at planting as basal application, and a second application was applied 3–4 weeks after sowing. For low N trials, all plots received recommended P (100 kg/ha) and/or K (50 kg/ha), followed by the suggested weed and insect control measures.

**TABLE 1:** Information on trials used for the genome-wide marker traits association study.

**Country**	**Location**	**Coordinates**	**Elevation (masl)**	**Management**	**Years/seasons**
Kenya	Kiboko	−2.250, 37.730	990	Optimum	2011A, 2012A
				Low N	2011A, 2011B, 2012A
	Embu	−0.500, 37.450	1492	Low N	2011A, 2011B, 2012A
	Kibos	−0.070, 34.820	1184	Optimum	2012A
	Kitale	1.010, 35.000	1859	Optimum	2011A, 2011A
	Kakamega	0.270, 34.740	1526	Optimum	2011A, 2012B
				Low N	2011A
Mexico	Agua Fria	20.530, −97.430	90	Optimum	2012A
				Low N	2011A
	Tlatizapan	18.680, −99.130	940	Low N	2010A
South Africa	Cedara	−29.530, 30.280	1100	Optimum	2011B
				Low N	2011B
Zambia	GART-Lusaka	−14.170, 28.370	1173	Low N	2011B
Zimbabwe	Harare	−17.800, 31.050	1498	Low N	2010B, 2011B

*A, main season; B, off-season; masl, meters above sea level; Low N, managed low N stress site.*

Data were collected for GY, anthesis date (AD), anthesis silking interval (ASI), plant height (PH), ear height (EH), ear position (EPO), ears per plant (EPP), and senescence (SEN). GY was calculated from field weight by adjusting the grain moisture to 12.5% and a shelling percentage of 80%. AD was recorded as the number of days from planting to when 50% of the plants in the plot started shedding pollen on the main axis of the tassel. ASI was calculated as the difference between the number of days when in 50% of the plants in a plot with 2–3 cm silk emerged and pollen shedding occurred. PH and EH were measured in centimeters as a distance from the base of a plant to the first branch of the tassel and the upper most ear from 10 representative plants, respectively. EPO was calculated as the ratio between PH and EH. SEN was recorded by visual assessment using a 1 to 10 scale, where 1 indicates that all leaves of all plants in a plot were green and 10 indicates that all leaves were dead. At harvest, edge plants were removed from all rows from trials planted under low N to avoid border effects.

Analyses of variance for each and across environments under each management condition were determined by the restricted maximum likelihood (REML) method using the R program embedded in META-R software (Alvarado et al., 2015). Experiments in the same location at different years were treated as different environments. All variance components were determined by the following linear mixed model:

$$Y_{i\,j\,k\,o}=\mu +L_{j}+R_{k}\,\left(L_{j}\right)+B_{0}\,\left[R_{k}\,\left(L_{j}\right)\right]+G_{i}+G\,L_{i\,j}+\varepsilon_{i\,j\,k\,o}$$

where *Y*_*ijko*_ is the phenotypic performance of the *i*^*th*^ genotype at the *j*^*th*^ environment in the *k*^*th*^ replication of the *o*^*th*^ incomplete block, μ was an intercept term, *G*_*i*_ was the genetic effect of the *i*^*th*^ genotype, *L*_*j*_ was the effect of the *j*^*th*^ environment, *GL*_*ij*_ was the interaction effect between genotype × environment, *R*_*k*_(*L*_*j*_) was the effect of the *k*^*th*^ replication within the *j*^*th*^ environment, *B*_0_[*R*_*k*_(*L*_*j*_)] is the random effect of incomplete block *o* within replicate *k* and location *j* and is assumed to be independently and identically normally distributed with mean zero and variance $\sigma_{B}^{2}\,\left(R\,L\right)$, and ε_*ijko*_ is the random residual error assumed independent and identically normally distributed with mean zero and variance σ_ε_^2^.

All effects are treated as random except for the effects of replications, which were treated as fixed. Heritability on an entry-mean basis was estimated from the calculated variance components as the ratio of genotypic to phenotypic variance. Further, best linear unbiased prediction (BLUP) and best linear unbiased estimation (BLUE) of each entry across environments within each management were calculated for all the traits. BLUEs were used for GWAS and GS analyses.

### DNA Extraction and Genotyping

With all the inbred lines in the IMAS association panel, genomic DNA was extracted from young leaves collected in a bulk of 10 plants per entry using a modified version of the CIMMYT high-throughput mini-prep Cetyl Trimethyl Ammonium Bromide (CTAB) method (Semagn, 2014). DNA samples were genotyped at the Institute of Biotechnology at Cornell University^1^, United States using *Ape*KI as the restriction enzyme and 96-plex multiplexing (Elshire et al., 2011). Raw GBS data for a total of 955,120 SNP loci distributed across the 10 maize chromosomes were received from the Institute of Genomic Diversity (IGD), Cornell University, United States. Different filtering criteria were applied to the raw data to get input data for LD analyses and GWAS. For LD, the raw data were filtered based on no missing data and >10% minor allele frequency (MAF). For GWAS, the genotype data were filtered with MAF of >5% and a minimum count of SNPs on 90% of the sample size using the Trait Analysis by Association, Evolution, and Linkage (TASSEL v.5.2.24) software (Bradbury et al., 2007).

### Population Structure and Linkage Disequilibrium

Population structure in the current association panel was investigated using classical multidimensional scaling (principal coordinate analysis) embedded in TASSEL v.5.2.24 software (Bradbury et al., 2007). TASSEL was also used for the analysis of kinship, genetic distances (Identity by state—IBS), and principle component analyses (PCAs).

Genome-wide and chromosome-specific LDs were estimated as a squared allele frequency correlation coefficient (*r*^2^) between all possible pairs of SNPs using TASSEL v5.2.31 (Bradbury et al., 2007). For genome-wide LD, 4,479 SNPs distributed across the 10 chromosomes, filtered based on no missing data per marker and >10% of MAF, were used. For chromosome-specific LD estimation, the SNPs were filtered with no missing data per marker and >1% of MAF. Full matrix LD analysis was performed with no imputation for missing data and setting heterozygous calls to missing. After analysis, all LD estimates with a missing value for distance were removed, and only LD estimates having *P* < 0.001 were considered significant (Pasam et al., 2012) and used for further analysis. LD decay rates were estimated by plotting localized regression curves (LOESS) of the *r*^2^ values versus the corresponding physical distances between the SNP pairs, followed by observation of the intersection point between the fitted LOESS curve and critical *r*^2^ values (Breseghello and Sorrells, 2006). For estimating LD decays within and across chromosomes, two background critical *r*^2^ values were considered for comparison. For the first baseline, critical *r*^2^ was determined by taking the parametric 95th percentile of the distribution of *r*^2^ values for unlinked SNPs. SNPs on different chromosomes and SNPs beyond 50 Mbp apart on the same chromosome are treated as unlinked (Pasam et al., 2012). The second baseline *r*^2^ value was 0.2, an arbitrary value often used to describe LD decay (Flint-Garcia et al., 2003; Zhu et al., 2008). Estimated LD decays were plotted in scatter plots and fitted with smooth curves by using LOESS function in R (R Core Team, 2016).

### Genome-Wide Association Analysis

Genome-wide association study analysis was done with the R package “FarmCPU—Fixed and random model Circulating Probability Unification” (Liu et al., 2016). The minimum input data required to run FarmCPU are genotypic data (GD), phenotypic data (Y), and genotypic map (GM) data. It takes genotypic data in numerical format; the “hmp” format was converted to numeric (0, 1, 2) using the “GAPIT” package (Lipka et al., 2012). The first three PCs obtained from TASSEL (Bradbury et al., 2007) were used as an input for GWAS in FarmCPU. The kinship was calculated with the default kinship algorithm in FarmCPU. The analysis was performed with maxLoop of five, a *p* threshold of 0.01, a QTN threshold of 0.01, and a MAF threshold of 0.05. The maxLoop refers to the total number of iterations used. The *p* threshold, QTN threshold, and MAF threshold refer to the *p* values selected into the model for the first iteration, the *p*-value selected into the model from the second iteration, and the minimum MAF of SNPs used in the analysis, respectively. For the *p*-values threshold, 0.01 refers to the Bonferroni threshold (0.01/number of the total markers used). In addition, the Bonferroni test threshold (0.01/number of markers) was used to set a significant level in Manhattan plots. “FarmCPU” also uses the “GAPIT” function to produce results, such as the Manhattan plot, the quantile–quantile (QQ) plot, GWAS results, and a marker effects table of user-provided covariates. Further, identifying putative genes in LD with significant SNPs and studying the function of those genes are useful for selecting significant SNPs to integrate into breeding programs. Putative genes were searched on the ensemble^2^ and maize gdb^3^ websites.

### Genomic Prediction

For the GP analysis, BLUEs across locations were used. Ridge-regression BLUP (RR-BLUP) with fivefold cross-validation was applied. From the GBS data, a subset of 5,929 SNPs with no missing values, distributed uniformly across the genome, and minor allele frequency > 0.04 were used for GP in an association panel. Details of the RR-BLUP model and its implementation are described by Zhao et al. (2012). With respect to the prediction accuracy for lines in the testing set, two GP approaches were evaluated: (1) “within-population and within-management” prediction, where lines within the association mapping panel were sampled to form both a training set and a testing set, and prediction was carried out within optimum and low N stress management; (2) “within-population and across-management” prediction, where trait data for the training set are sampled from optimum management condition and a testing population is sampled from low N stress conditions. The second approach is mimicking the indirect selection for GY for low N stress conditions. The prediction accuracy was calculated as the correlation between the observed phenotypes and genomic estimated breeding values (GEBVs) divided by the square root of the specific trait heritability (Dekkers, 2007). Sampling of the training and testing sets was repeated 100 times for each approach.

## Results

### Phenotypic Data

**FIGURE 1:**
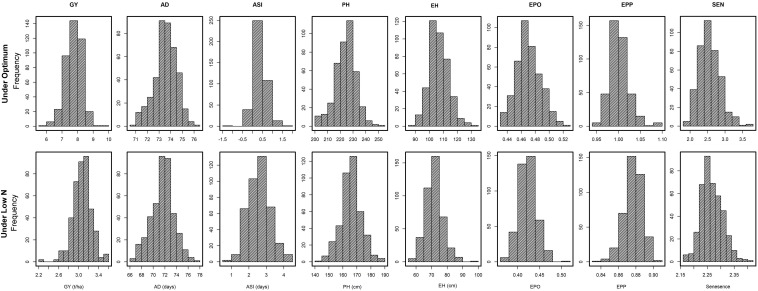
Phenotypic distribution for eight tested traits tested in multiple locations under optimum and low N conditions. *GY*, grain yield; *AD*, days to anthesis; *ASI*, anthesis-silking interval; *PH*, plant height; *EH*, ear height; *EPO*, ear position, *EPP*, ears per plant; and *SEN*, senescence.

The frequency distribution and descriptive statistics for GY and other traits revealed a wide variation for both optimum and low N stress conditions (Figure 1). The variation for GY ranged from 5.7 to 9.5 t/ha (mean = 7.74 t/ha) under optimum and from 2.2 to 3.6 t/ha (mean = 3.1 t/ha) under low N stress (Table 2). The mean performance for AD showed 2 days earliness in low N stress compared to optimum conditions. The range is higher for ASI under low N stress compared to optimum conditions. The means of PH and EH were reduced significantly under low N stress compared to optimum conditions. Further, the range of distribution reduced drastically for SEN under low N stress compared to optimum conditions.

**TABLE 2:** Quantitative genetic parameters for testcross progenies of IMAS association mapping panel evaluated in nine locations under optimum and 13 locations under low N conditions.

	**GY**	**AD**	**ASI**	**PH**	**EH**	**EPO**	**EPP**	**SEN**
**Optimum**								

	7.74	73.6	0.39	224.2	106.8	0.47	1.01	2.56
Mean (range)	(5.79 to 9.54)	(70.6 to 76.0)	(−1.14 to 1.77)	(200.4 to 253.5)	(87.7 to 132.7)	(0.43 to 0.53)	(0.95 to 1.10)	(1.56 to 4.16)
σ^2^_*G*_	0.42**	1.25**	0.19**	80.66**	55.20**	0.001*	0.001**	0.14**
σ^2^_*GxE*_	0.67**	0.90**	0.001*	27.19**	14.99**	0.001*	0.003**	0.12**
σ^2^_*E*_	3.02**	142.10**	0.47**	711.61**	121.17**	0.002**	0.007**	0.17**
σ^2^_*e*_	1.61	4.28	2.36	131.20	79.18	0.001	0.018	0.20
*h*^2^	0.72	0.79	0.60	0.89	0.90	0.84	0.48	0.85
LSD	2.49	4.06	3.01	22.45	17.44	0.07	0.26	0.87
CV	16.39	2.81	37.76	5.11	8.33	7.17	13.32	17.29

**Low N**								

	3.07	71.6	2.60	165.8	71.8	0.43	0.88	2.27
Mean (range)	(2.24 to 3.59)	(66.7 to 77.1)	(0.75 to 4.47)	(144.9 to 189.4)	(57.3 to 95.1)	(0.36 to 0.51)	(0.83 to 0.91)	(2.17 to 2.41)
σ^2^_*G*_	0.06**	3.99**	0.56**	62.47**	34.39**	0.001**	0.0001*	0.01**
σ^2^_*GxE*_	0.16**	1.01**	0.55**	17.12**	10.47**	0.0001*	0.001**	0.02**
σ^2^_*E*_	0.94**	69.79**	3.23**	1015.11**	369.05**	0.002**	0.007**	0.86**
σ^2^_*e*_	0.64	3.59	2.30	127.56	72.12	0.002	0.020	0.28
*h*^2^	0.64	0.95	0.81	0.91	0.91	0.88	0.33	0.38
LSD	1.57	3.71	2.97	22.14	16.65	0.07	0.27	1.04
CV	26.21	2.64	56.77	6.80	11.81	8.91	15.96	23.39

*^,^ **Significance at *P* < 0.05 and *P* < 0.01, respectively; σ^2^_*G*_, genotypic variance; σ^2^_*GxE*_, genotypic × environment interaction variance; σ^2^_*E*_, environmental variance; σ^2^_*e*_, error variance; h^2^, broad sense heritability. *GY* grain yield, *AD* days to anthesis, *ASI* anthesis-silking interval, *PH* plant height, *EH* ear height, *EPO* ear position, *EPP* ears per plant, and *SEN* senescence.

The analysis of variance indicated that the effects of genotype, environment, and their interaction are significant (*P* ≤ 0.05) for GY and other seven traits both under optimum and low N stress conditions (Table 2). The extent of variation was higher for GY, PH, EH, and SEN under optimum conditions, whereas for AD and ASI, the magnitude of variation was higher under low N stress conditions. The heritability estimates were moderate to high both under optimum and low N conditions.

**FIGURE 2:**
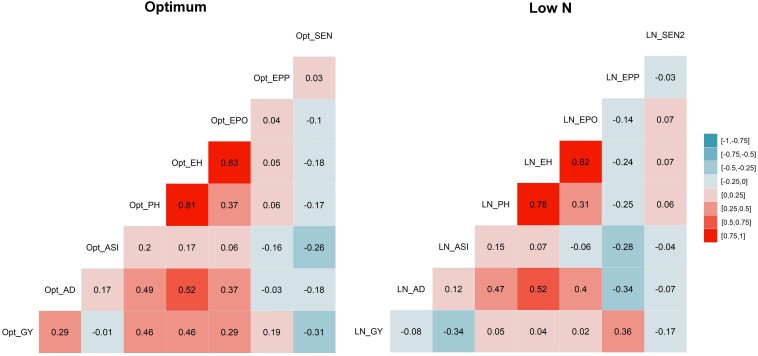
Correlations among eight tested traits. Correlation between traits under optimum condition are listed on the left, and those under low N condition are listed on the right. The correlation level is color-coded according to the color key plotted on the extreme right. Correlations with >?0.10 and >0.16 were significant at 0.05 and 0.01 levels, respectively. *GY*, grain yield; *AD*, days to anthesis; *ASI*, anthesis-silking interval; *PH*, plant height; *EH*, ear height; *EPO*, ear position; *EPP*, ears per plant; and *SEN*, senescence under optimum (Opt) and low N (LN) conditions.

The correlation coefficients illustrated significant positive correspondence of GY with AD, PH, EH, EPO, and EPP (*r* = 0.19–0.46) under optimum conditions, whereas they negatively correlated with ASI and no significant correlation was found with AD, PH, EH, and EPO under low N stress (Figure 2). Further, SEN was consistently significant and negatively correlated with GY under both managements, whereas it significantly and negatively correlated with AD, ASI, PH, and EH only under optimum condition.

### Summary of SNP and Inbred Lines

The summary of 182,252 SNPs used in this study is presented in Table 3. From 955,120 GBS SNPs used to genotype 411 inbred lines, only 19% were retained after filtering with the twin criteria of >5% MAF and <10% missing per marker. The number of markers retained ranged from 12,338 on chromosome 10 to 29,248 on chromosome 1. For all the retained markers, alleles with a frequency below 50% were considered minor. The percentage of missing markers per individual inbred line varied from 0 to 10%, and the overall average was 4.2%.

**TABLE 3:** The distribution of SNPs, percentage of missing markers, minor allele frequency, and heterozygous markers across the 10 maize chromosomes in diverse tropical maize inbred lines.

**Chr.**	**Raw Data**	**Filtered**	*Mean Distance	**Missing (%)**			**MAF**	**Heterozygous**		
				**Mean**	**Minimum**	**Maximum**	**Ave**	**Minimum**	**Maximum**	**Mean**
1	148752	29248	0.029	0.042	0.00	0.10	0.21	0.00	0.77	0.103
2	115173	22180	0.022	0.042	0.00	0.10	0.21	0.00	0.37	0.104
3	108224	20921	0.021	0.041	0.00	0.10	0.21	0.00	0.43	0.100
4	94726	17263	0.017	0.041	0.00	0.10	0.21	0.00	0.56	0.100
5	110328	21566	0.022	0.042	0.00	0.10	0.21	0.00	0.48	0.104
6	76475	14336	0.014	0.042	0.00	0.10	0.21	0.00	0.45	0.102
7	80517	15323	0.015	0.043	0.00	0.10	0.20	0.00	0.43	0.098
8	81431	15602	0.016	0.041	0.00	0.10	0.21	0.00	0.61	0.101
9	72368	13475	0.013	0.042	0.00	0.10	0.21	0.00	0.72	0.103
10	67126	12338	0.012	0.042	0.00	0.10	0.21	0.00	0.45	0.100
Total	955120	182252	0.018	0.042	0.00	0.10	0.21	0.00	0.53	0.101

*Mean distance between adjacent markers in Mbp; Chr., chromosome; MAF, minor allele frequency; min, minimum; max, maximum. Mean distance, missing, MAF, and heterozygosity are reported for SNPs after filtering 10% missing and 5% MAF.

The proportion of heterozygosity of SNPs (the number of taxa that are heterozygous for a given SNP divided by the total number of individuals) ranged from 0 to 0.77, with an overall average of 0.10. The minimum proportion of heterozygous SNPs was found on chromosome 2 and the maximum on chromosome 1. The heterozygosity of inbred lines (the number of heterozygous markers per inbred line divided by the total number of markers) ranged from 0.002 to 0.354 with an overall average of 0.103. About half of the inbred lines showed heterozygosity of less than 0.05, and 67% of the inbred lines had heterozygosity of less than 0.125.

### Population Structure, Kinship, and Genetic Distance

The 411 individuals in the current association panel showed moderate structure (Supplementary Figure S1), which is one of the possible causes for false-positive results during marker trait association analysis. In FarmCPU, the first three PCs are recommended to be added in the GWAS model to minimize the risk of false positives (Liu et al., 2016) arising from the population structure. Even though 79 PCs were required to explain 50% of the variance in the inbred lines, only 3 PCs (explaining > 10% of the variance) were included in the FarmCPU GWAS analysis. The FarmCPU method output includes the effects of user-provided PCs, which turned out to be small for all the traits analyzed.

Another important parameter that affects the GWAS is kinship among the tested inbred lines. About 99% of the pairwise kinship comparisons among 411 inbred lines had a value of <0.5, indicating the low relatedness among the inbred lines used for GWAS. The kinship heatmap (Supplementary Figure S2) generated using the vanRanden algorithm in the “GAPIT” basic scenario also indicated low levels of relatedness among most pairs of inbred lines. In the heatmap, the count of the kinship values reached a maximum at the value of zero, further confirming low levels of relatedness among the tested inbred lines. In addition, genetic distance among 84,255 pairwise comparisons ranged from 0.004 to 0.339 with an average of 0.311. The proportion of pairwise comparisons with values higher than 0.3 was 14.95% and with values higher than 0.2 was more than 99%, indicating the amount of genetic diversity in this association panel.

### Linkage Disequilibrium

The distance over which LD persists will control the marker number and density, as well as the appropriate experimental design to perform an association analysis (Flint-Garcia et al., 2003). The genome-wide and chromosome-specific LDs in this study were estimated at two critical *r*^2^ levels (*r*^2^ = 2.0 and *r*^2^ = 0.34; Table 4). In the genome-wide LD analysis, the *r*^2^ values for only 6% of the total pairwise comparisons were significant (*P* < 0.001). The proportion of significant *r*^2^ values for the 10 chromosomes were in the similar range (3–4%). Among the significant *r*^2^ values, the proportion with *r*^2^ > 0.2 was the lowest for genome-wide LD (3%) compared to individual chromosomes (ranging from 5 to 8% with an average of 6%).

**TABLE 4:** Genome-wide and chromosome wise LD decay at two critical *r*^2^ values (0.2 and 0.34).

**Chr**	**No of SNPs**	**No. of Pairwise Comparisons**	***r*^2^ (%)**			**LD Decay**	
			***P* < 0.001**	**>0.2**	**Avg**	***r*^2^ = 0.2**	***r*^2^ = 0.34**
1	1261	794430	3	5	0.09	0.23	0.14
2	979	478731	3	5	0.09	0.20	0.09
3	1003	502503	4	6	0.09	0.17	0.11
4	781	304590	3	7	0.10	0.22	0.07
5	885	391170	3	6	0.09	0.20	0.12
6	618	190653	3	5	0.09	0.17	0.04
7	695	241165	4	5	0.09	0.25	0.10
8	717	256686	4	6	0.09	0.26	0.09
9	576	165600	4	8	0.11	0.34	0.18
10	659	216811	4	8	0.11	0.13	0.10
GW	4479	10028481	6	3	0.08	0.24	0.19

*Chr., chromosome; GW, genome-wide; LD, linkage disequilibrium; Avg., average.*

Genome-wide LD decays at *r*^2^ = 0.20 and *r*^2^ = 0.34 were 0.24 and 0.19 Mbp, respectively (Supplementary Figure S3). Chromosome-specific LD decays ranged from 0.13 to 0.34 Mbps with an average of 0.22 Mbp at the critical *r*^2^ = 0.20 and ranged from 0.04 to 0.18 Mbps with an average of 0.10 Mbp. At *r*^2^ = 0.34, LD decay was fastest for chromosome 10 (0.13 Mbp) and extended for chromosome 9 (0.34 Mbp). At *r*^2^ = 0.34, the LD decayed fast for chromosome 6 (0.04 Mbp) and again delayed for chromosome 9 (0.18). The LD decay at the arbitrary *r*^2^ = 0.2 was less variable than the LD decay at the calculated *r*^2^ = 0.34.

### Genome-Wide Marker Traits Association Analyses

The Q–Q plot of the FarmCPU model resulted in a sharp deviation from the expected *P*-value distribution in the tail area, demonstrating that false positives and negatives were adequately controlled (Supplementary Figure S4). GWAS analyses revealed 38 and 45 significant SNPs under optimum and low N conditions, respectively, at 5% Bonferroni significance level (*P* < 2.7 × 10^–7^, Tables 5–7 and Supplementary Figure S4). For all eight traits, the number of significant SNPs dropped to 33 under optimum and 27 under low N conditions when a stringent Bonferroni 1% significance level (*P* < 5.4 × 10^–8^) was used. The distribution of significant SNPs across chromosomes varied between 2 in chromosome 9 and 15 in chromosome 1 at a Bonferroni threshold of 5% and ranged from 2 (chromosome 9) to 12 (chromosome 1) at a Bonferroni threshold of 1%. The MAF for significant SNPs ranged between 8 and 35% at a Bonferroni threshold of *P* < 1% and between 13 and 35% at a Bonferroni threshold of *P* < 5%.

**TABLE 5:** Number of markers significantly associated with grain yield and other traits at 5 and 1% Bonferroni threshold levels.

**Trait**	**Bonferroni Threshold (Low N)**		**Bonferroni Threshold (Optimum)**		**Total**
	***P* < 0.01**	***P* < 0.05**	***P* < 0.01**	***P* < 0.05**	
GY	5	6	4	5	11
AD	7	8	4	6	14
ASI	4	7	10	11	18
EH	9	10	1	2	12
EPO	5	6	3	4	10
EPP	1	4	–	–	4
PH	2	4	2	5	9
SEN	–	–	3	5	5
Total	33	45	27	38	83

*GY, grain yield; *AD*, days to anthesis; *ASI*, anthesis-silking interval; *PH*, plant height; *EH*, ear height; *EPO*, ear position; *EPP*, ears per plant, and *SEN*, senescence.*

Association analyses for GY identified five significant SNPs under optimum condition and six significant SNPs under low N condition at a Bonferroni 5% threshold. The allelic effect (difference in mean performance for GY between testcross hybrids with major allele and minor allele) for these significant SNPs ranged from −0.16 to 0.39 under optimum conditions and −0.10 to 0.11 under low N conditions. A positive value indicates that the minor allele was the favorable allele associated with the increase in GY, and a negative value indicates that the major allele was the favorable allele associated with GY. The significant SNPs for GY were found on chromosomes 1, 2, 4, 5, 7, 8, and 10 with the most significant one being located on chromosome 10 (*P* = 1.78 × 10^–12^). Chromosomes 4, 8, and 10 had SNPs identified only under optimum conditions, while chromosomes 1 and 7 housed SNPs detected only under low N conditions. Chromosomes 2 and 5 hosted SNPs identified under both optimum and low N conditions. Information on all the significant SNPs, their corresponding MAF, and the allelic effect are listed in Table 6.

**TABLE 6:** List of significant SNPs associated with GY and AD under optimum and low N managements using the FarmCPU model with a Bonferroni threshold of *P* < 0.05.

**Trait**	**SNP**	**Chr**	**Position(bp)**	***P* value**	**MAF**	**Effect**
GY_Opt	S2_144477756	2	144477756	1.62E-07	0.09	0.23
	S4_178469568	4	178469568	2.49E-08	0.37	–0.16
	S5_183614607	5	183614607	7.31E-11	0.06	0.39
	S8_75414416	8	75414416	2.29E-09	0.21	0.23
	S10_147459915	10	147459915	1.78E-12	0.08	0.35
GY_LowN	S1_25425465	1	25425465	8.51E-08	0.14	–0.09
	S1_202550249	1	202550249	4.40E-08	0.40	–0.07
	S2_107767802	2	107767802	7.25E-10	0.15	0.11
	S5_152923661	5	152923661	3.33E-08	0.12	–0.10
	S5_214168220	5	214168220	2.35E-07	0.34	–0.07
	S7_128740455	7	128740455	1.57E-09	0.12	0.11
AD_Opt	S2_142865720	2	142865720	2.19E-07	0.32	–0.22
	S2_210662089	2	210662089	4.87E-08	0.15	0.30
	S3_149683417	3	149683417	1.75E-09	0.11	–0.40
	S6_102939532	6	102939532	2.51E-07	0.05	0.46
	S7_156476052	7	156476052	6.10E-14	0.41	0.35
	S8_170275834	8	170275834	9.43E-13	0.41	0.31
AD_LowN	S1_283191977	1	283191977	2.36E-08	0.09	0.57
	S2_131348717	2	131348717	2.43E-10	0.20	–0.49
	S2_210662089	2	210662089	1.77E-08	0.15	0.45
	S4_170809248	4	170809248	3.78E-10	0.27	–0.49
	S6_150843360	6	150843360	4.26E-09	0.06	0.76
	S7_123828656	7	123828656	4.87E-08	0.45	–0.37
	S10_126930458	10	126930458	1.79E-11	0.08	–0.76
	S10_147898784	10	147898784	2.68E-07	0.19	–0.40

*Where MAF is the Minor Allele Frequency, effect is the Allele Effect, and *P*-value is for the mixed linear model, GY grain yield, and *AD* days to anthesis under optimum (Opt) and low N stress (Low N) conditions.*

For AD, a total of 13 significant SNPs, 6 under optimum and 8 under low N, were detected across all chromosomes except chromosomes 5 and 9 (Table 6). One SNP on chromosome 2 (*S2_210662089*; *P* = 1.77E-08 under low N; *P* = 4.87E-08 under optimum) was common between optimum and low N conditions. SNPs on chromosomes 2, 6, and 7 were detected under both optimum and low N conditions, whereas all the other SNPs were specific to either low N or optimum conditions. The allelic effect for these significant SNPs ranged from −0.40 to 0.46 under optimum conditions and −0.76 to 0.76 under low N conditions. The largest numbers of significant SNPs were identified for ASI followed by AD. For ASI, 18 significant SNPs were distributed across all 10 chromosomes (Table 7). Chromosome 6 had an SNP detected under low N, whereas chromosomes 5, 7, 8, and 9 had SNPs associated with ASI only for optimum conditions. All other chromosomes carried SNPs for both optimum and low N conditions. The SNP on chromosome 5 was the most significant in the current study (*P* = 4.59E-14). Two SNPs on chromosome 3 (*S3_147401613* for ASI under low N and *S3_149683417* for AD under optimum) and another two SNPs on chromosome 7 (*S7_155590511* for ASI and *S7_156476052* for AD; both under optimum) were located a few Mbps away from each other. The allelic effect for these significant SNPs ranged from −0.21 to 0.37 under optimum conditions and −0.16 to 0.17 under low N conditions (Table 7).

**TABLE 7:** Details of the significant SNPs associated with ASI, Pht, Eht, EPO, EPP, and SEN under optimum and low N managements using the FarmCPU model with a Bonferroni threshold of *P* < 0.05.

**Trait**	**SNP**	**Chr.**	**Position**	***P*-value**	**MAF**	**Effect**
ASI_Opt	S1_274946693	1	274946693	2.08E-07	0.08	–0.21
	S2_6636633	2	6636633	4.99E-09	0.17	0.17
	S2_54204647	2	54204647	2.82E-08	0.44	0.11
	S3_128687310	3	128687310	8.68E-10	0.33	0.16
	S4_235073935	4	235073935	4.22E-08	0.06	0.22
	S5_195672028	5	195672028	4.59E-14	0.07	0.37
	S7_24409023	7	24409023	3.88E-12	0.25	0.18
	S7_155590511	7	155590511	8.19E-08	0.22	0.12
	S8_136094451	8	136094451	5.01E-10	0.26	–0.14
	S9_118046290	9	118046290	7.10E-08	0.22	0.13
	S10_33353122	10	33353122	2.32E-09	0.16	–0.20
ASI_LowN	S1_5810155	1	5810155	3.93E-08	0.19	0.17
	S2_226325975	2	226325975	1.54E-07	0.29	–0.15
	S3_32033690	3	32033690	1.58E-07	0.49	0.13
	S3_147401613	3	147401613	1.06E-08	0.43	–10.15
	S4_37297564	4	37297564	1.68E-07	0.22	–0.16
	S6_164497574	6	164497574	9.27E-09	0.27	0.16
	S10_4586049	10	4586049	6.45E-09	0.48	–0.16
Pht_Opt	S3_64819581	3	64819581	1.62E-07	0.06	3.46
	S4_46166070	4	46166070	7.98E-08	0.37	–1.69
	S4_184955101	4	184955101	3.98E-08	0.25	2.10
	S7_6297685	7	6297685	5.22E-12	0.26	–2.77
	S10_143502717	10	143502717	1.61E-07	0.16	2.19
Pht_LowN	S1_17679579	1	17679579	4.65E-08	0.10	2.32
	S3_199254673	3	199254673	1.88E-13	0.21	–3.54
	S4_237693358	4	237693358	2.59E-07	0.12	2.50
	S8_25351243	8	25351243	1.05E-07	0.32	–1.48
Eht_Opt	S2_140662928	2	140662928	2.44E-07	0.27	–1.71
	S8_158098622	8	158098622	7.18E-09	0.37	1.75
Eht_LowN	S1_16698847	1	16698847	2.94E-12	0.12	–2.55
	S1_199339693	1	199339693	1.60E-08	0.06	1.92
	S1_274946693	1	274946693	3.84E-09	0.08	–2.31
	S2_196870448	2	196870448	1.54E-08	0.11	1.63
	S3_217796834	3	217796834	7.05E-08	0.15	1.37
	S5_83133270	5	83133270	5.66E-10	0.06	2.60
	S6_7046560	6	7046560	4.63E-09	0.24	–1.26
	S7_40379325	7	40379325	4.01E-08	0.21	1.23
	S10_126687226	10	126687226	1.81E-10	0.07	2.24
	S10_145097517	10	145097517	2.10E-08	0.33	1.17
EPO_Opt	S1_71065792	1	71065792	1.76E-07	0.07	0.01
	S6_97945994	6	97945994	1.47E-08	0.09	0.01
	S8_72067641	8	72067641	2.88E-09	0.37	0.00
	S10_143712477	10	143712477	2.71E-08	0.08	0.01
EPO_LowN	S1_207055175	1	207055175	1.37E-07	0.22	0.00
	S1_274946693	1	274946693	3.01E-09	0.08	–0.01
	S1_285229689	1	285229689	2.52E-10	0.42	–0.01
	S2_33350339	2	33350339	8.02E-10	0.33	0.00
	S6_97945994	6	97945994	4.75E-11	0.09	0.01
	S10_123956017	10	123956017	6.75E-09	0.13	0.01
EPP_LowN	S1_122756821	1	122756821	2.54E-08	0.47	–0.01
	S4_174009677	4	174009677	1.17E-07	0.07	–0.02
	S5_188825516	5	188825516	1.01E-07	0.18	–0.01
	S10_148304779	10	148304779	1.33E-07	0.31	0.01
SEN_Opt	S1_220067760	1	220067760	3.46E-09	0.47	–0.08
	S4_177150249	4	177150249	1.53E-08	0.19	0.10
	S5_8351127	5	8351127	1.02E-07	0.45	–0.08
	S8_159648136	8	159648136	2.12E-07	0.24	–0.08
	S9_153449703	9	153449703	1.70E-08	0.47	–0.08

*Where MAF is the Minor Allele Frequency, effect is the Allele Effect, and *P*-value is for the mixed linear model’ *ASI*, anthesis-silking interval; *PH*, plant height; *EH*, ear height; *EPO*, ear position; *EPP*, ears per plant; and *SEN*, senescence under optimum (Opt), and low N stress (Low N) conditions.*

PH, EH, and EPO are interrelated agronomic traits in maize. At the 5% Bonferroni level, 9, 12, and 10 significant SNPs were detected for PH, EH, and EPO, respectively. Among these SNPs, 4, 10, and 6 were associated with PH, EH, and EPO, respectively, specifically under low N conditions. An SNP on chromosome 6 (*S6_97945994*; *P* = 4.75E-11 under low N and *P* = 1.47E-08 under optimum) was common between optimum and low N conditions for EPO. SNPs significantly associated with the target trait under both optimum and low N managements were found on chromosomes 3 and 4 for PH; chromosome 2 for EH; and chromosomes 1, 6, and 10 for EPO. Other chromosomes harbored SNPs detected only under one management condition. Under optimum conditions, the allelic effect of significant SNPs for PH ranged from −2.77 to 3.46; for EH, the range varied from −1.71 to 1.75; whereas for EPO, the allele effect was small. On the contrary, under low N conditions, the allelic effect of significant SNPs for PH ranged from −3.54 to 2.50; for EH, the range varied from −2.55 to 2.60; whereas for EPO, the allele effects range was small.

EPP had four significant SNPs, all under low N conditions. These SNPs were distributed across chromosomes 1, 4, 5, and 10. The SNPs for EPP on chromosomes 4, 5, and 10 were situated close to the SNPs identified for GY under optimum conditions. The allelic effect for these significant SNPs was very small and ranged between −0.02 and 0.01 under low N conditions (Table 7). Unlike all other traits investigated in this study, significant SNPs for SEN were detected only under optimum N conditions with allele effects ranging from −0.08 to 0.10.

The availability of common markers for multiple traits is crucial for simultaneous improvement of two or more traits. In this study, a common SNP was identified for EPO and EH under low N conditions on chromosome 1 (*S1_274946693*; *P* = 3.01E-09). In addition, there were a few closely linked SNPs on chromosome 1 that are associated with EPO and EH under low N conditions; and PH and EPO under optimum and EH under low N conditions.

Based on the physical positions of the significantly associated SNPs, a total of 56 and 80 genes^4^ assumed to be the potential candidate genes for GY and other traits were found for optimum and low N conditions, respectively. The list of these 136 genes and their corresponding functional annotations is provided in Supplementary Table S1. Seven SNPs were linked with four known genes. Fertilization Independent Endosperm 1 (*FIE1*) and Teosinte Glume Architecture *(TGA1*) genes were in LD with *S4_37297564* and *S4_46166070*, respectively on chromosome 4. *FIE1* was associated with ASI under low N and *TGA1* was associated with PH under optimum conditions.

**FIGURE 3:**
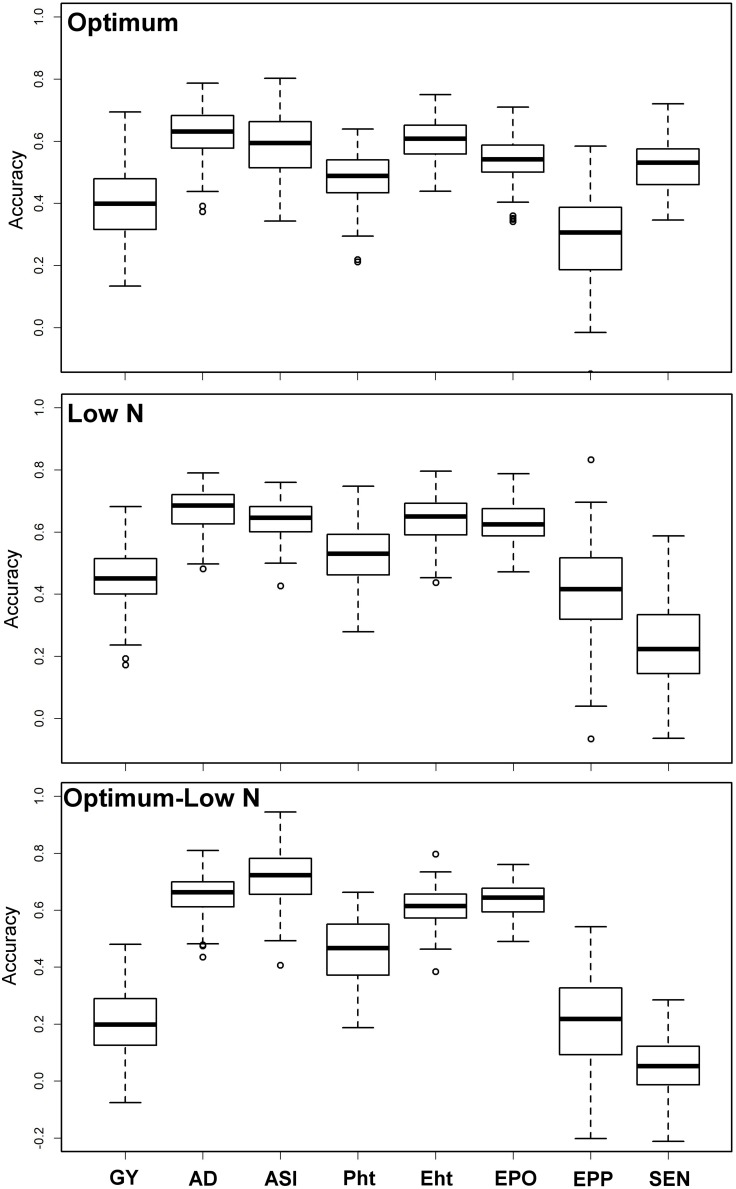
Prediction accuracy for BLUEs estimated across locations for eight tested traits under optimum and low N conditions using genome-wide SNPs. Optimum—training and estimation set within optimum condition, Low N—training and estimation set is within low N condition, Optimum–Low N—training is based on optimum conditions and estimation is under low N conditions. *GY*, grain yield; *AD*, days to anthesis; *ASI*, anthesis-silking interval; *PH*, plant height; *EH*, ear height; *EPO*, ear position; *EPP*, ears per plant; and *SEN*, senescence.

Prediction accuracies were moderate to high for all eight traits under both optimum and low N conditions (Figure 3). The observed prediction accuracy for GY, AD, ASI, PH, EH, EPO, EPP, and SEN were 0.42, 0.62, 0.59, 0.48, 0.60, 0.54, 0.29, and 0.52, respectively, under optimum conditions and 0.45, 0.67, 0.64, 0.53, 0.64, 0.63, 0.42, and 0.24, respectively, under low N conditions. Prediction accuracies were slightly decreased when the training populations were derived from optimum conditions and used to predict the performance of the same trait/s under low N conditions. The observed prediction accuracy for GY was decreased drastically to 0.20, whereas for other traits like AD, ASI, PH, EH, EPO, EPP, and SEN, the accuracies were 0.65, 0.71, 0.46, 0.61, 0.64, 0.20, and 0.06, respectively.

## Discussion

In SSA, smallholder farmers face difficulty in following the recommended dose of fertilizer application to harvest the real yield potential. Understanding GY and its related traits performance under low N soil will be beneficial for the development of low N stress resilient maize varieties. However, accurate and consistent phenotyping under low N condition is challenging and highly prejudiced by genotype, environment, soil, management, and their interactions. Therefore, integration of modern breeding tools with conventional breeding not only improves the understanding on the genetic basis of traits performance under low N but also helps in developing the improved cultivars with high yielding and stable performance.

Adequate variation at the phenotypic level and a high level of polymorphisms at the DNA sequence level are vital factors for high-quality genetic mapping (Yan et al., 2009). Phenotypic data under low N conditions usually have low heritability due to the inherent variability in low N stressed fields. In this study, extensive genetic variance with moderate to high heritability estimates and high genetic variance were observed under both optimum and low N conditions. We found significant genotypic and environmental variation for GY and other agronomic traits. Several researchers also reported similar significant variations in response to abiotic stresses (Betrán et al., 2003; Worku et al., 2007; Derera et al., 2008; Badu-Apraku et al., 2015; Ribeiro et al., 2018). The high significant GXE observed under low N conditions for all traits agrees with the findings of Makumbi et al. (2011), Ertiro (2018), and Ribeiro et al. (2018). The high heritability estimates recorded for most measured traits under optimum and low N conditions indicated that the expression of these traits was consistent. Most of the traits had substantially high heritability estimates indicating their stability in the expression, and further, they also are highly correlated with GY, which can be used in indirect selection for increasing GY under each environment. These results are consistent with the findings of Ertiro (2018) and Ribeiro et al. (2018). Relatively small experimental errors in this study were attributed to the use of many locations (9 optimum and 13 low N) with appropriate experimental designs, which effectively estimated main quantitative genetic factors associated with the traits. As a result, the mean phenotypic data of most traits were normally distributed presenting an ideal dataset for genome-wide marker-trait association study (Table 2 and Figure 1).

The correlations between GY and other agronomic traits were changed with management conditions (Figure 2). Under the optimum condition, GY was significant and positively correlated with AD, PH, EH, EPO, and EPP, but stayed independent with ASI. Whereas under low N conditions, these correlations decreased drastically, like significant but negatively correlated with ASI and no significant correlation with PH, EH, and EPO. This must be considered while breeding for GY under low N, as other yield-related traits may not have similar correlations as we expected under optimum conditions. EPP was significant and negatively correlated with AD, ASI, PH, EH, and EPO. SEN was significant and negatively correlated with GY and other traits except EPP.

A marker–trait association study was performed for the eight traits, which were evaluated under optimum and managed low N stressed conditions. Several factors influence the GWAS results including, but not limited to, the quality of phenotypic data, the complexity of the trait genetic architecture, the extent of genetic diversity in the germplasm, and LD relationships between causal variants and genotyped SNPs (Flint-Garcia et al., 2003; Zhu et al., 2008; Soto-Cerda and Cloutier, 2010; Scherer and Christensen, 2016). However, factors affecting the accuracy of GWAS could be improved through appropriate experimental designs and statistical packages.

The set of lines used in this study was assembled from different tropical breeding programs within the CIMMYT global maize program and national agricultural research systems (NARS) in Africa, and these lines were bred for tolerance to various biotic and abiotic stresses (Gowda et al., 2015). It is not unexpected to get high genetic distance and lower kinship among the inbred lines; hence, stratification of the inbred lines into different groups based on the breeding goals and adaptation is expected. Standard GWAS test statistics assume that all materials included for the analysis are unrelated and selected from uniform, random-mating population. Any departure from these assumptions can cause unanticipated results (Scherer and Christensen, 2016) leading to spurious associations due to false positives. Use of appropriate statistical analysis that accounts for family relatedness and population structure is crucial in order to avoid the occurrence of these false positives.

The extent of LD in a set of germplasm affects the mapping resolution and the number of markers required for association mapping studies (Yu and Buckler, 2006). LD is further affected by the extent of genetic diversity captured by the population under study (Soto-Cerda and Cloutier, 2010). Genome-wide and chromosome-specific LD decays in this study were extended over a few hundred kilobases. Genome-wide LD decay was 230 kb at *r*^2^ = 0.2 and was 190 kb at *r*^2^ = 0.34. For individual chromosomes, this value was in the range of 130–340 kb at *r*^2^ = 0.2 and 40–180 kb at *r*^2^ = 0.34. Gowda et al. (2015) also found similar results for a subset of this association panel used in their study. Previous studies on maize showed rapid LD decay (1 kb) in landraces, approximately 2 kb in diverse inbred lines, and up to several hundred kilobases in commercial elite inbred lines (Jung et al., 2004). The relatively high LD in the current study is due to the inclusion of elite inbred lines assembled from tropical breeding programs within CIMMYT and NARS (Gowda et al., 2015). Based on the observed LD, significant marker–trait associations can be identified using a moderate to high number of markers (Yan et al., 2011).

Taking family relatedness and population structure into consideration, FarmCPU identified several SNPs associated with the causative variant for each trait under optimum and low N conditions. Out of 83 SNP–trait associations declared significant at a Bonferroni 5% threshold, three SNPs on chromosomes 1, 2, and 6 were associated either with different traits or different management conditions for the same trait, suggesting pleiotropic effects of genes associated with these significant markers. Common SNPs under optimum and low N conditions would be useful for simultaneous improvement of trait/s for both optimum and low N stress conditions.

In addition to discovering SNPs significantly associated with traits, identifying putative genes in LD with significant SNPs and studying the function of the genes and the biological pathways in which the putative genes participate (Scherer and Christensen, 2016) are crucial for using these SNPs in breeding programs. Putative genes were searched on the maize gdb^5^ and ensemble^6^ websites. The *FIE1* gene, likely to have acquired important novel functions for endosperm development and its maternal alleles, gets activated 2 days after pollination (Hermon et al., 2007) indicating its role in ASI. Narrow ASI is one of the desirable secondary traits that is significantly correlated with high GY under stress conditions. The marker linked to the gene could be used for selecting genotypes having favorable alleles for narrow ASI.

Teosinte glume architecture1 (TGA1) is another key gene in the evolution of teosinte that exposed the kernel on the surface of the ear on modern maize (Wang et al., 2005), which showed strong association with SNPs identified for PH. Assaying the border effects of TGA1 reduced or eliminated the TGA1 gene expression using the RNAi (ribonucleic acid interference) construct. On several branching and kernel traits, maize lines expressing an RNAi construct targeting TGA1 displayed pleiotropic morphological effects (Wang et al., 2015). With regards to branching, these RNAi lines probably remove the repressive function of TGA1/neighbor of tag1 (NOT1), allowing the outgrowth of axillary branches. Both TGA1 and NOT1 belong to the SQUAMOSA promoter binding proteins (SBP) family of transcription factors. Members of this family have been shown to regulate development of meristem and also play a role in producing both plant architecture and ear phenotypes (Chuck et al., 2010, 2014). The presence of *TGA1/NOT1* in duplication may have facilitated its subfunctionalization. For example, *TGA1* alone controls the fruit case/cob, whereas *TGA1* functions in a redundant manner with *NOT1* to regulate plant architecture traits. So, the SNP marker associated with *TGA1* is useful for MAS.

Another SNP (*S4_237693358*) on chromosome 4 associated with PH under low N conditions was linked with three gene models, namely, *Zm00001d053632*, *Zm00001d053633*, and *Zm00001d053635*. These genes models were in LD with a known gene “RPS8,” ribosomal protein S8. RPS8 belongs to the 40S ribosomal protein S8 family. This gene was associated with PH under low N conditions, which indicates that rps8 genes might have a role in the control of PH under stress conditions. Another SNP “*S7_123828656*” on chromosome 7 was associated with AD under low N conditions and was in LD with seven gene models, of which two (*Zm00001d020584* and *Zm00001d020585*) were associated with a known histone H4 gene (*H4C7*). The gene belongs to the histone H4 protein family.

Successful integration of modern tools helped to achieve high genetic gain for complex traits in maize breeding (Beyene et al., 2019; Yuan et al., 2019). With rapid progress in genotyping technology and statistical models, genomic prediction of breeding value has been successfully applied in maize for quantitative traits (Zhang et al., 2015, 2017; Beyene et al., 2019; Yuan et al., 2019). In the present study, we compared the prediction accuracies under optimum and low N conditions (Figure 3). Interestingly, the prediction accuracies were slightly higher for GY, AD, ASI, EPO, and EPP under low N conditions compared to optimum conditions, whereas for PH, EH, and SEN, the accuracy was higher under optimum conditions. The accuracy observed for all traits under both optimum and low N conditions reveals the effect of heritability as the traits with higher heritability generally had higher prediction accuracy. The observed prediction accuracies for GY and other traits are comparable to earlier studies reported under different stresses in maize (Zhang et al., 2015; Beyene et al., 2019; Yuan et al., 2019). In GS, the less complex trait AD and ASI had higher accuracy compared to GY, which is consistent with the nature of trait complexity (Zhang et al., 2017; Yuan et al., 2019).

Breeding for low N stress tolerance is complex and very expensive. Several studies were reported on the efficiency of indirect selection to improve GY for low N by using secondary traits and/or GY under optimum conditions (Ertiro, 2018). With similar assumption, here we tried to use a training population based on traits data under optimum conditions and predict the same trait performance under low N conditions. The observed prediction accuracy for GY is 0.20 (Figure 3), far below than the phenotypic selection efficiency, which is 0.41 (the square root of heritability of GY) under low N stress. However, with three cycles per year and the requirement of lesser resources for implementing GS compared to phenotypic selection, GS is endorsed to integrate to improve the selection efficiency for low N stress as an attractive option in a long-run objective of the breeding program. For other traits, accuracies are relatively high, which clearly supports the usefulness of GS in their improvement under either optimum or stress condition.

GWAS results revealed that NUE in maize is controlled by numerous genes with minor effects, which are seriously influenced by environmental factors and thus are difficult to track effectively by conventional breeding alone. High throughput and large-scale routine phenotypic evaluation are still far from implementation for several NARS breeding programs. GS in combination with mapping approaches like GWAS and QTL mapping can enhance the efficiency to improve the trait of interest. Integration of GS with GWAS results leads not only to the increase in the prediction accuracy but also helps in validating the function of the identified candidate genes as well as the increase in the accumulation of favorable alleles with minor and major effects. Further, GS can remarkably reduce the resources required for selection and can improve breeding efficiency, especially when genotyping is very cheap, and several commercial service providers are available. With this kind of progress, one can expect that GS can integrate the powerful GWAS results from previous studies and projects and used them in breeding maize with high NUE.

## Conclusion

Through GWAS, marker–trait associations were detected for GY and other traits measured under low N and optimum conditions. Eighty-three significant marker–trait associations were identified for all the traits under both optimum and low N conditions. No common markers were identified for GY between optimum and low N conditions, confirming that a different genetic mechanism for GY under optimum and low N conditions is a possibility. The result further confirms higher efficiency of direct selection in target environments for the improvement of GY. For some secondary traits, common markers were obtained under both management conditions, suggesting the likelihood of simultaneous improvement for two or more secondary traits using the same set of markers. The physical position of significant markers coincided with 136 putative protein coding genes. Four known genes were associated with traits under optimum and low N conditions. Markers associated with these known genes could be used in breeding for the improvement of the associated traits. Prediction accuracy for all traits under both optimum and low N conditions is promising. Further, incorporating the trait-associated markers detected through GWAS into the prediction model has a potential to improve the prediction accuracy for the quantitative traits like GY under stress conditions, which leads to significant improvement for NUE in maize.

## Data Availability Statement

All datasets generated for this study are included in the article/Supplementary Material.

## Author Contributions

BE, ML, MO, BD, BP, and MG contributed in the project planning and overall coordination. BE performed and coordinated the field experiments. MG and BE were responsible for coordinating sample management and genotyping. BE, ML, BD, and MG carried out the analysis. BE, ML, BP, and MG wrote the manuscript. All authors have made their contribution in editing the manuscript and approved the final version.

## Conflict of Interest

The authors declare that the research was conducted in the absence of any commercial or financial relationships that could be construed as a potential conflict of interest.

## Footnotes

1 http://www.biotech.cornell.edu/brc/genomics-facility

2 http://plants.ensembl.org/biomart/martview/ce35c2dc12e78418361fb4cffa43bdbe

3 http://www.maizegdb.org/

4 http://plants.ensembl.org/Zea_mays/Gene/Summary

5 http://www.maizegdb.org/

6 http://plants.ensembl.org/biomart/martview
