# Supplementary material for: Genetic Dissection of Nitrogen Use Efficiency in Tropical Maize Through Genome-Wide Association and Genomic Prediction
Source: Front Plant Sci. 2020 Apr 28;11:474. doi: 10.3389/fpls.2020.00474 (PMC7198882; doi:10.3389/fpls.2020.00474)
Supplement: Supplementary file 2 [file Data_Sheet_2.docx]

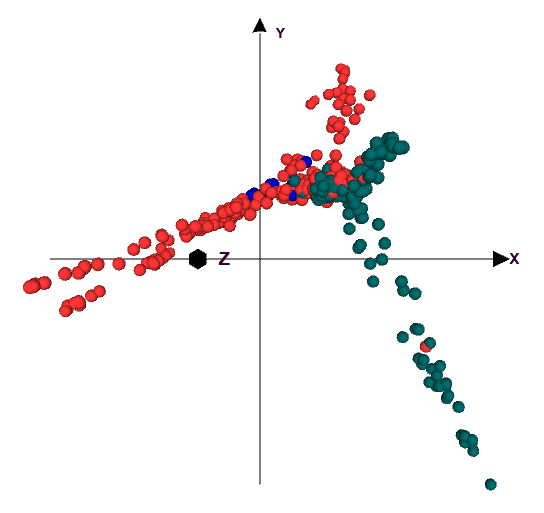


**Supplementary Figure S1**. Principal component analysis for 411 individuals with 182,252 GBS SNP markers. Lines were grouped based on adaptation as highland (blue color), mid-altitude level (orange color) and low land (green color) maize lines.


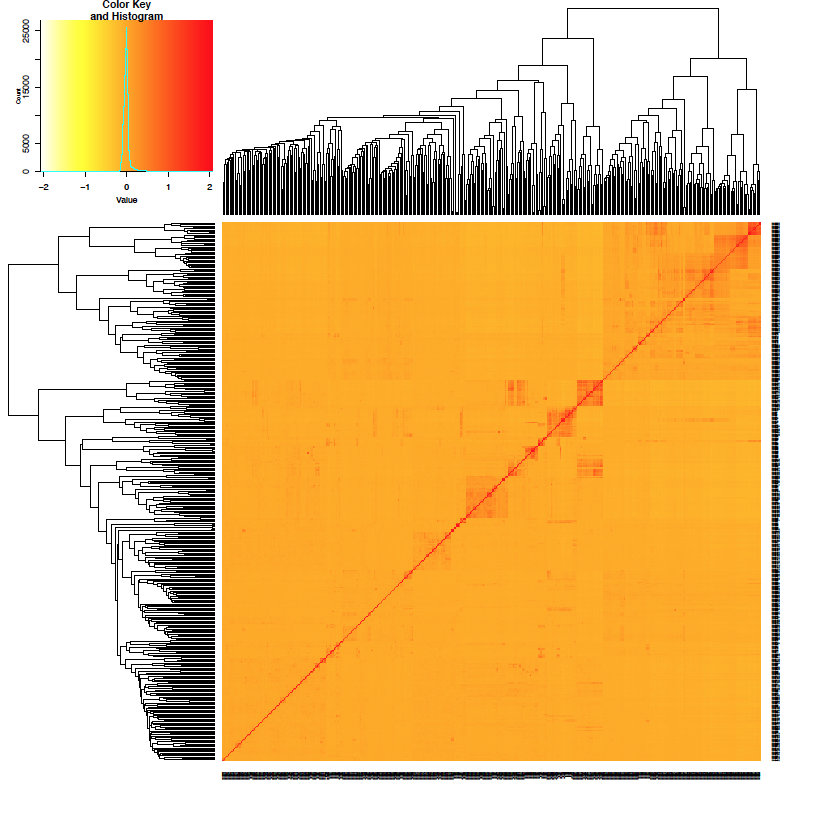


**Supplementary Figure S2**. Kinship heatmap generated for 411 inbred lines from 182,252 GBS SNP markers


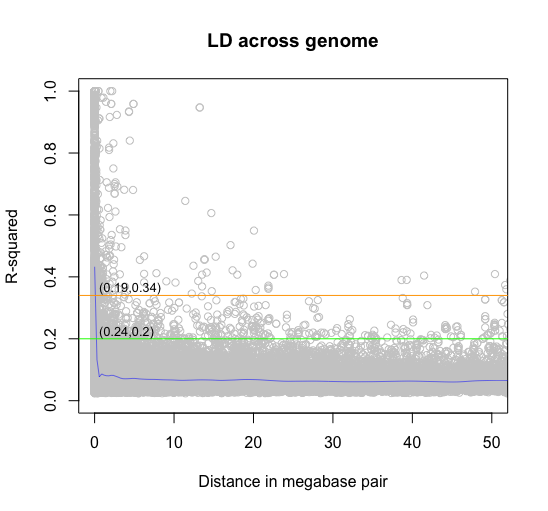

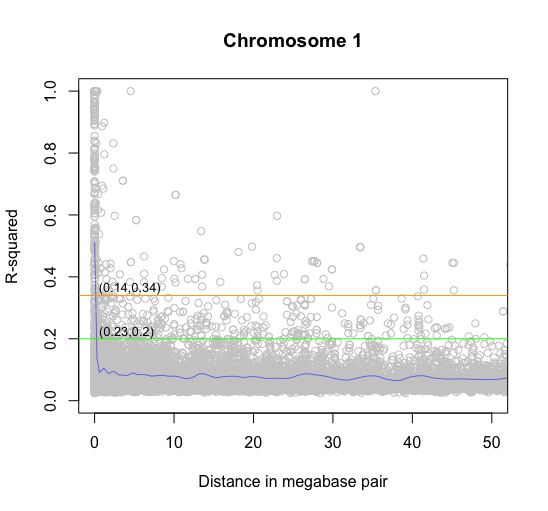

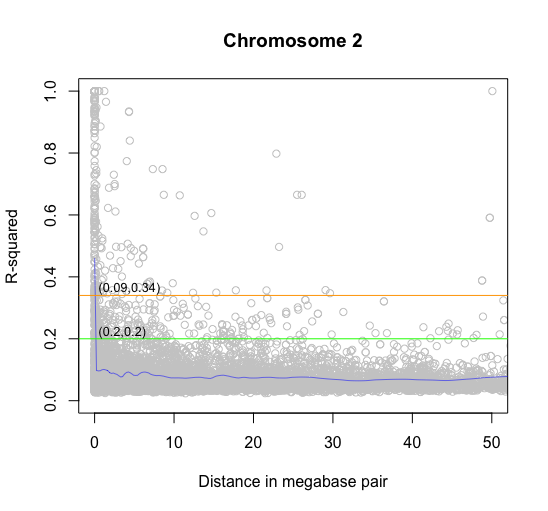

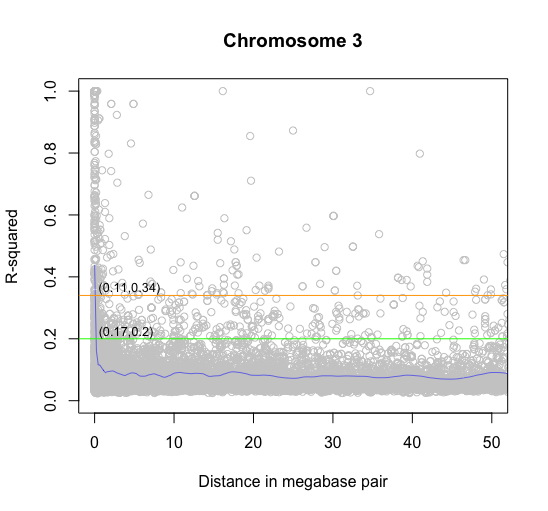

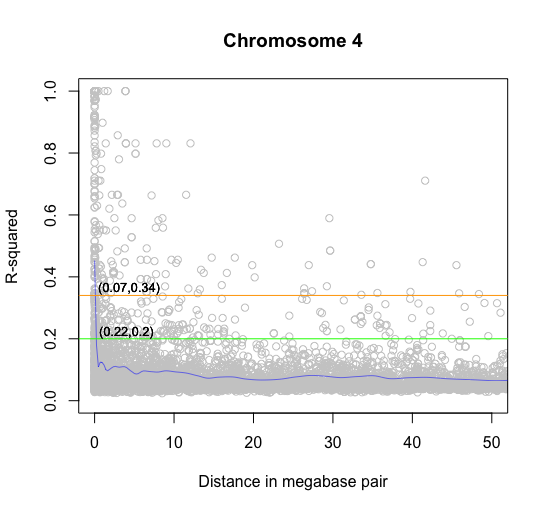

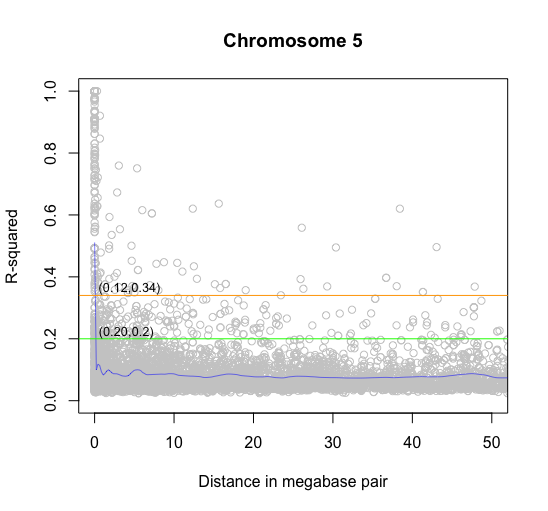


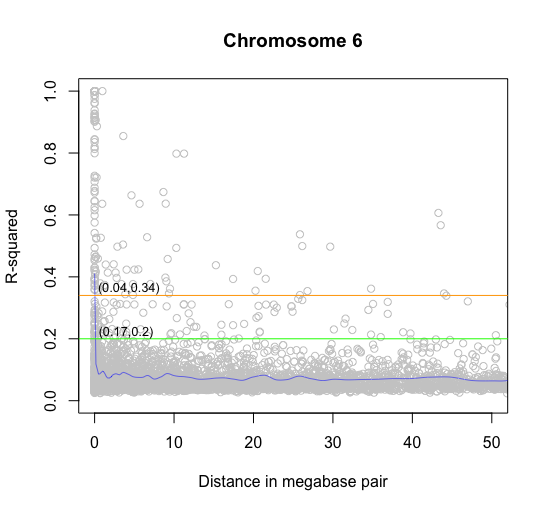

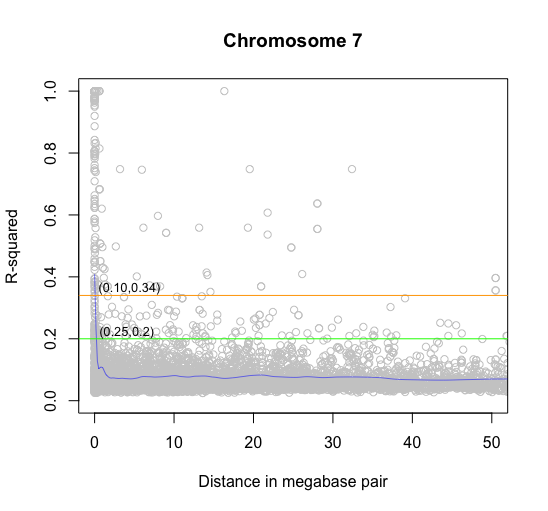

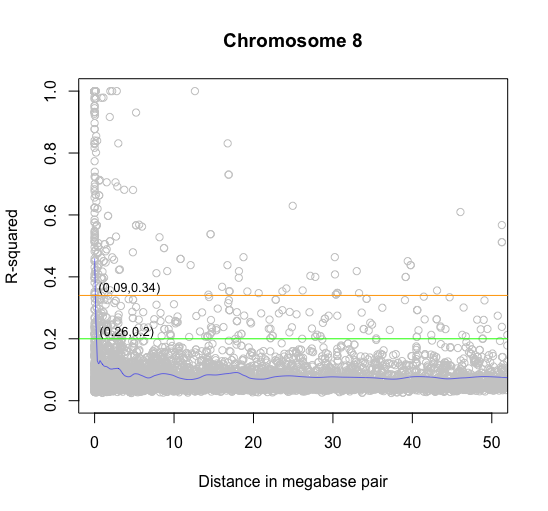

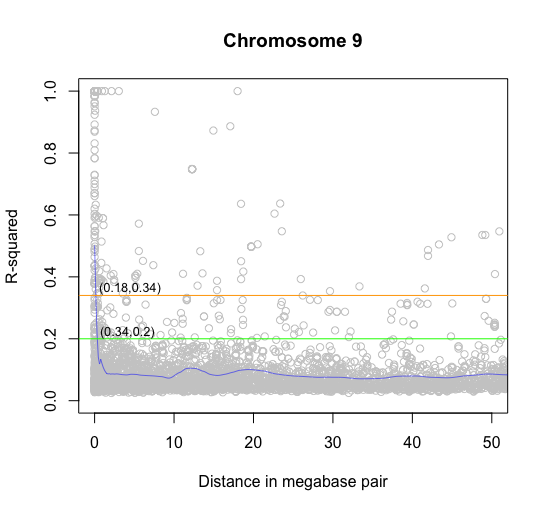

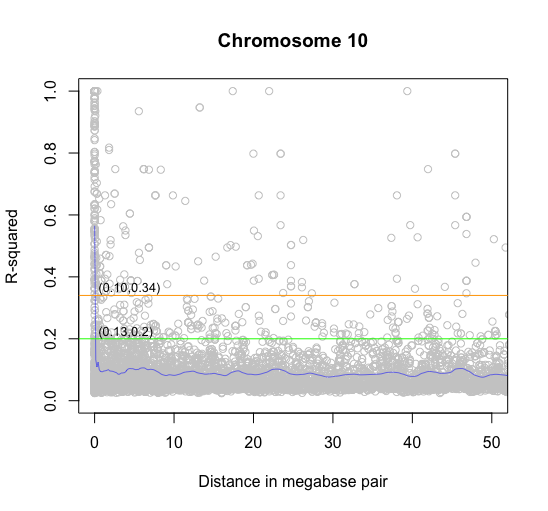


**Supplementary Figure S3**. Genome-wide and chromosome specific LD decay plots at two cutoff points (green line, r^2^=0.2 (arbitrary r^2^ value) and orange line, r^2^=0.34 (Calculated r^2^ value))
