# Supplementary material for: Genetic Dissection of Nitrogen Use Efficiency in Tropical Maize Through Genome-Wide Association and Genomic Prediction
Source: Front Plant Sci. 2020 Apr 28;11:474. doi: 10.3389/fpls.2020.00474 (PMC7198882; doi:10.3389/fpls.2020.00474)
Supplement: Supplementary file 3 [file Data_Sheet_3.zip › Supplementary Figure S4.docx]

Optimum Low N


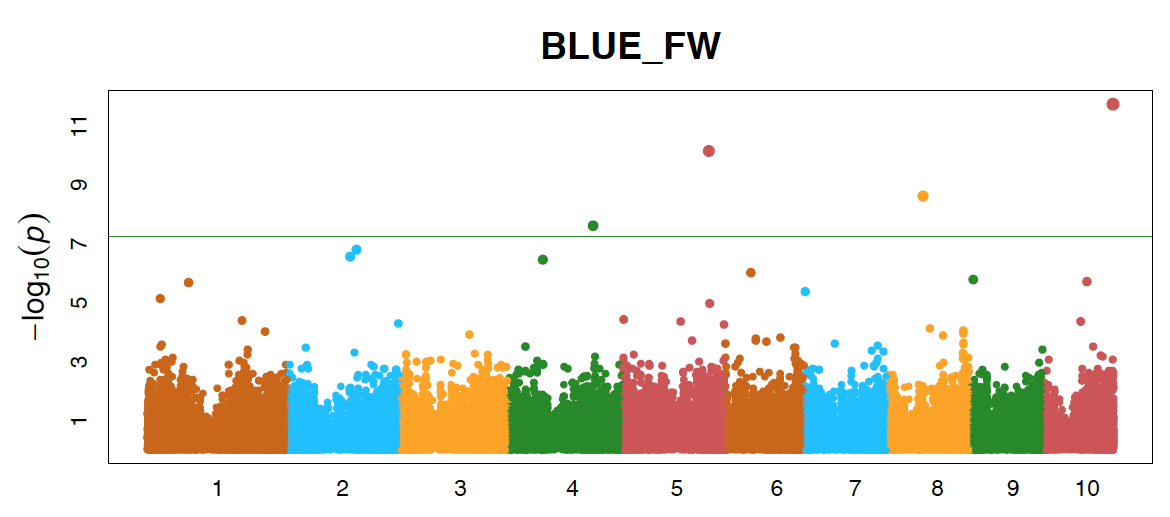

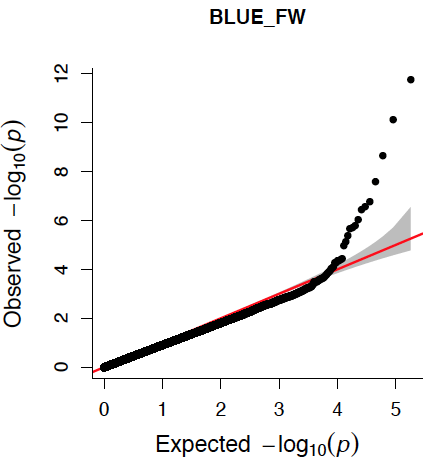

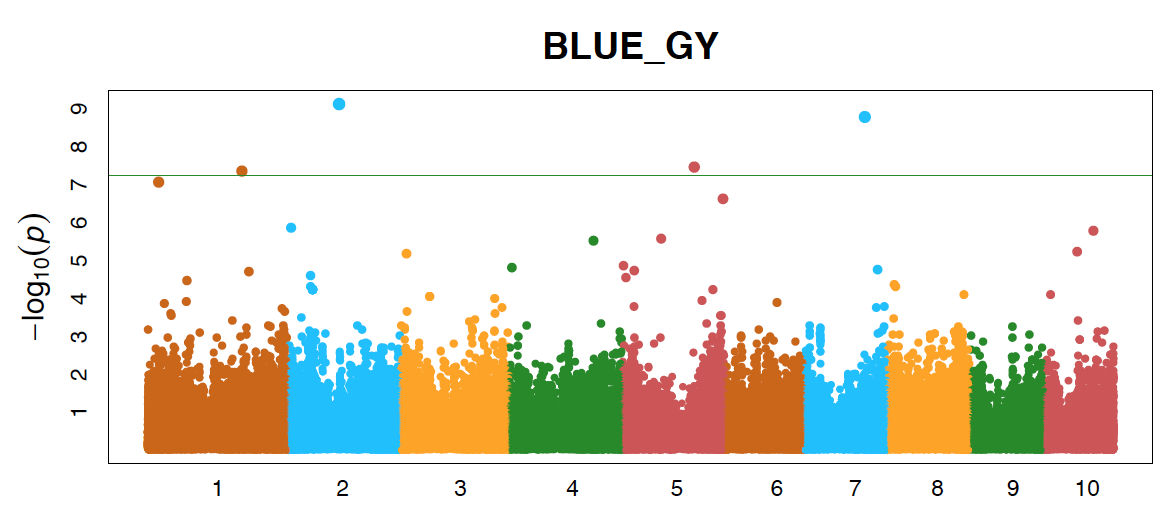

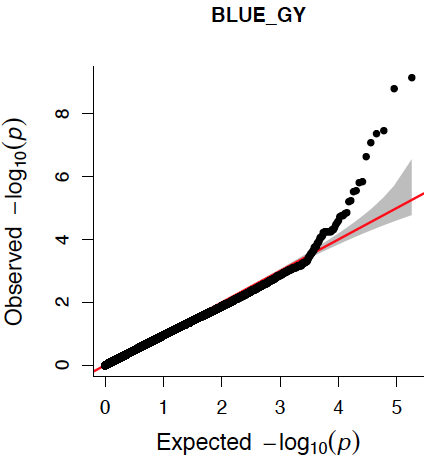


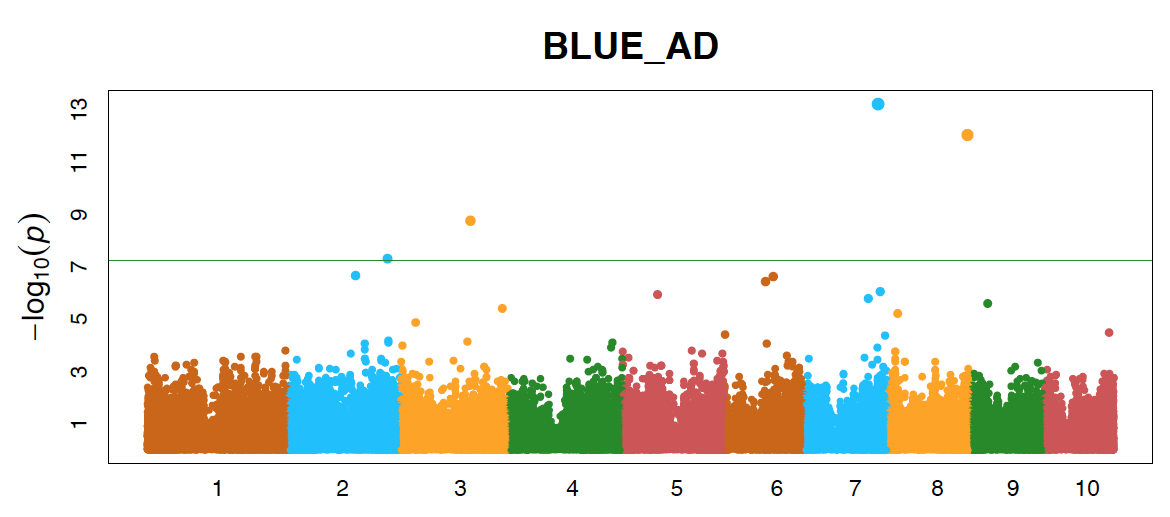

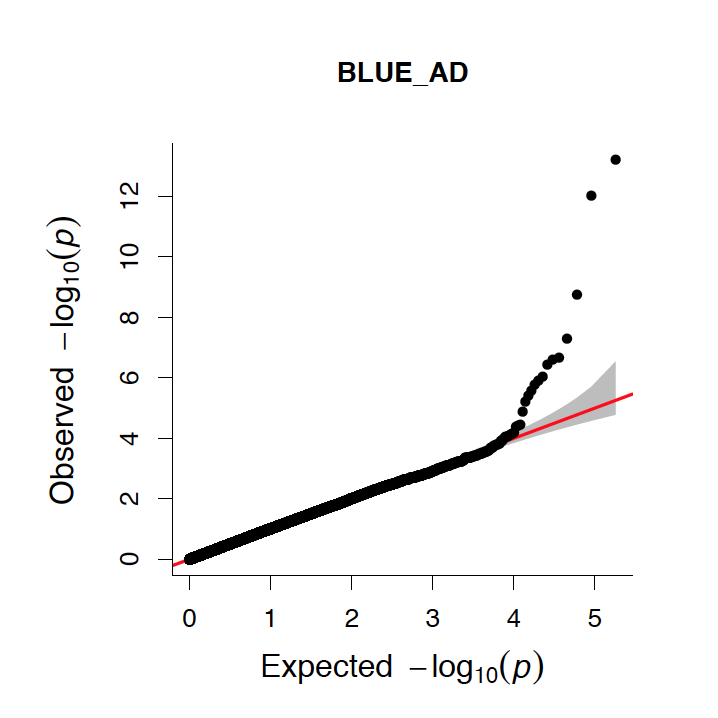

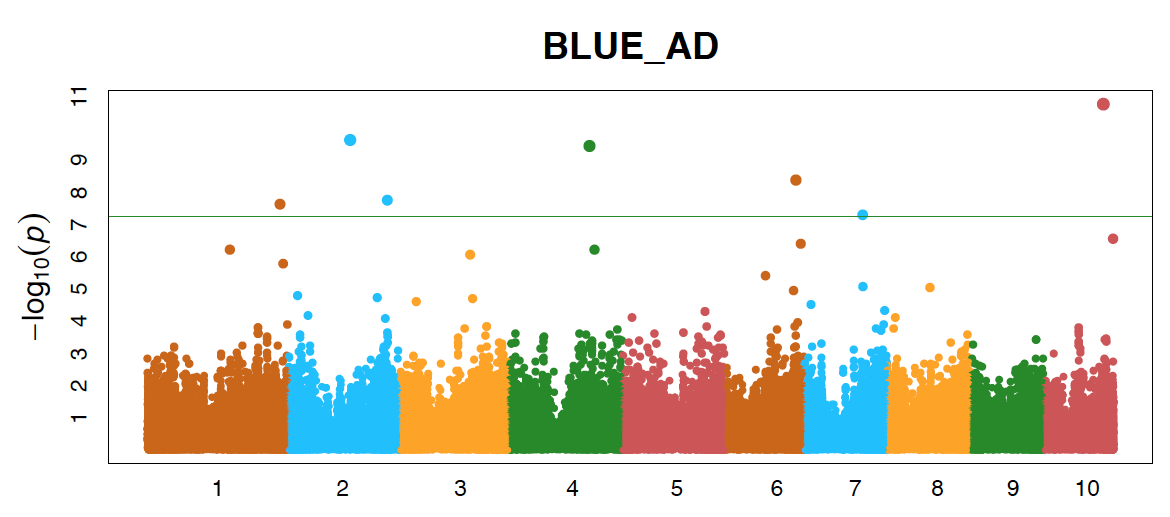

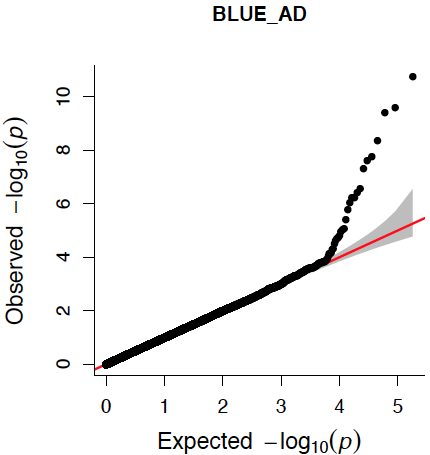


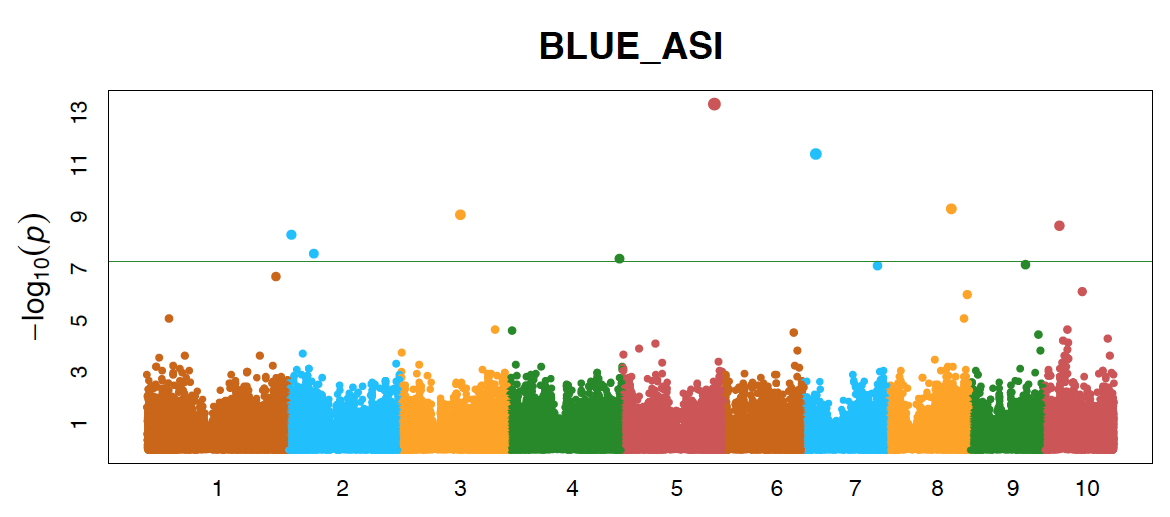

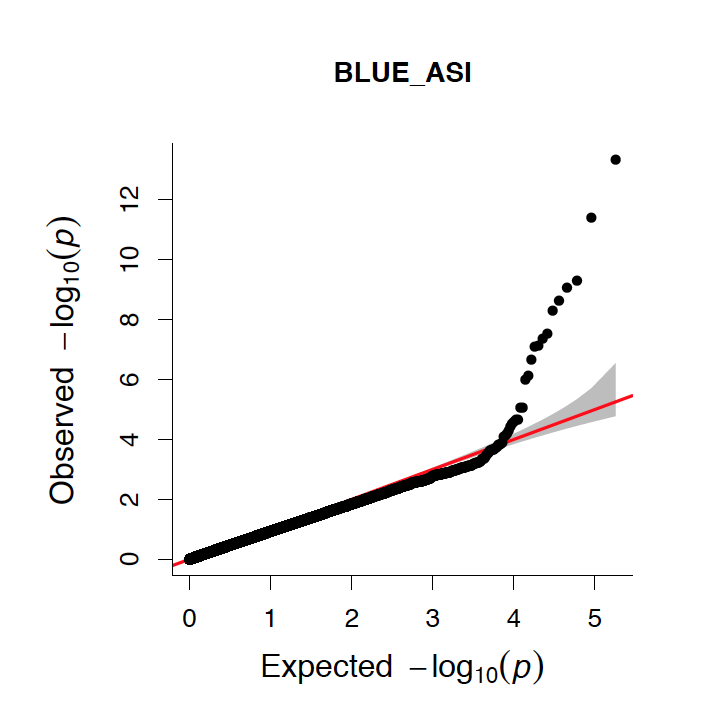

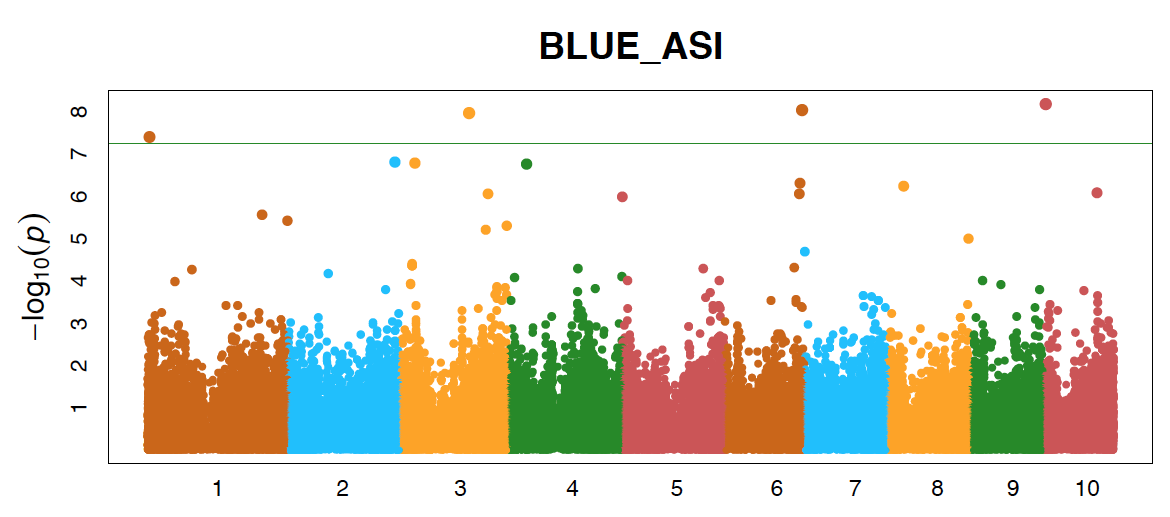

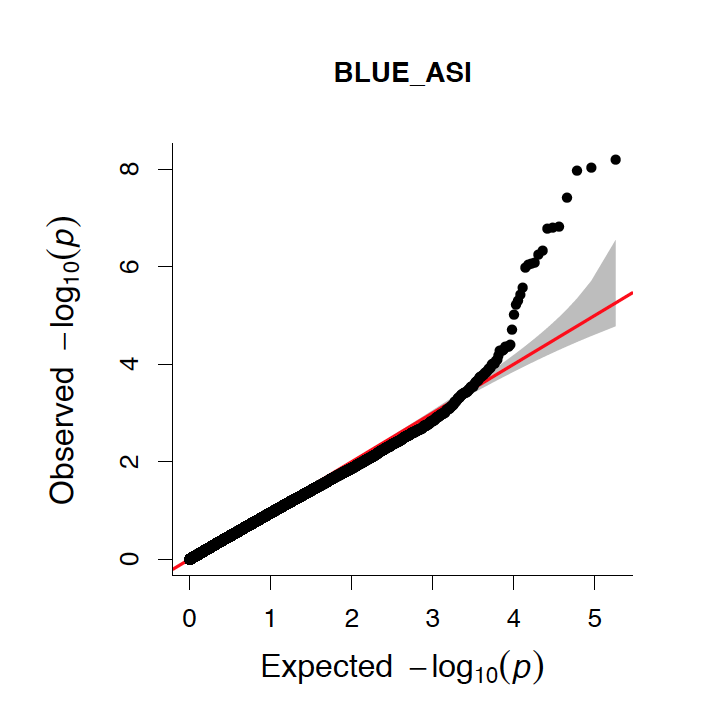


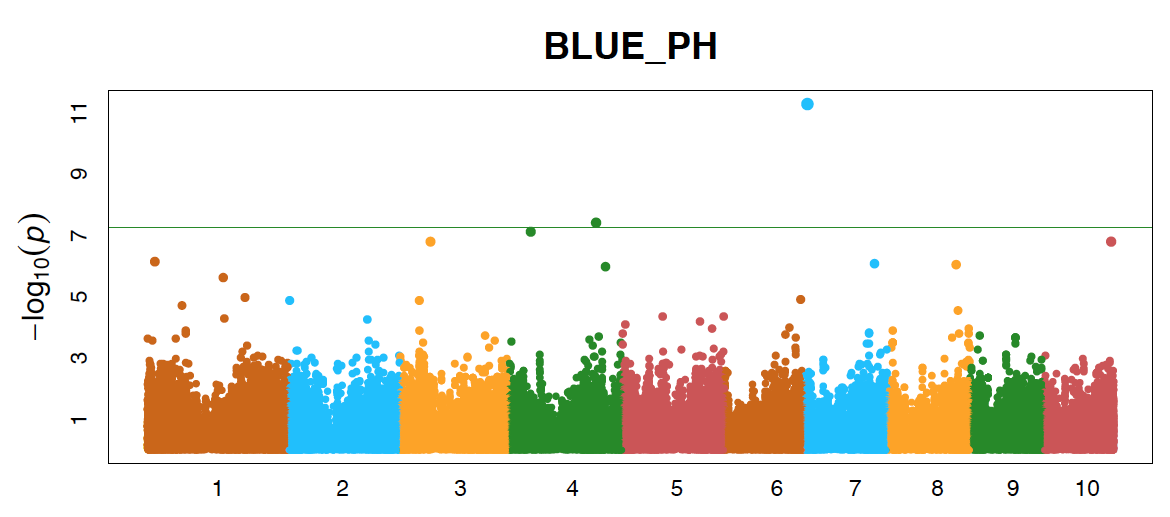

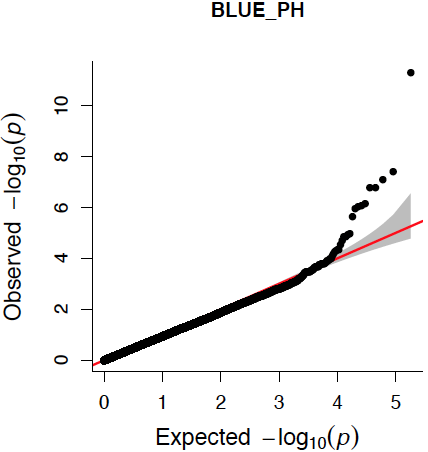

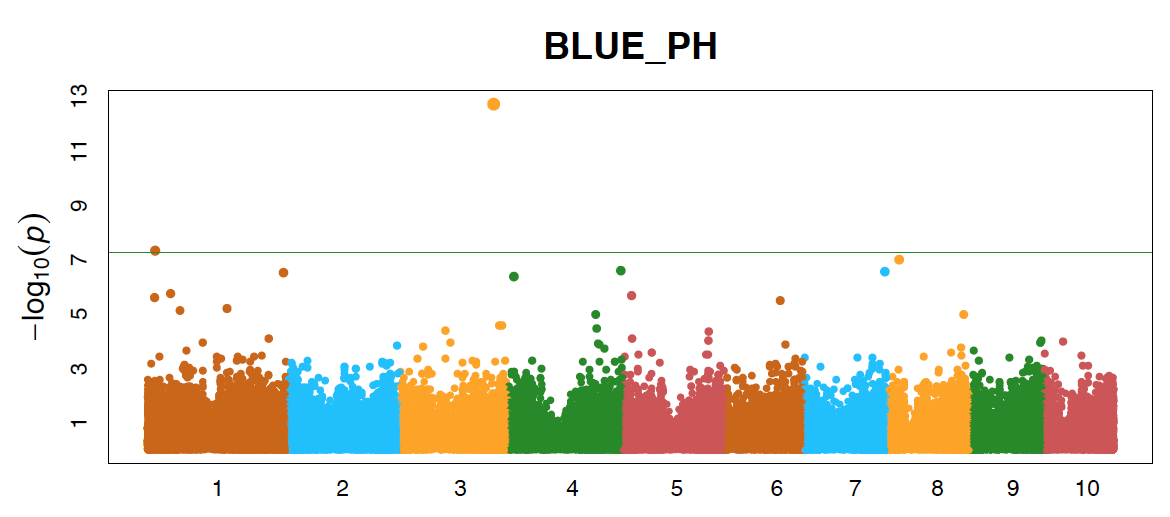

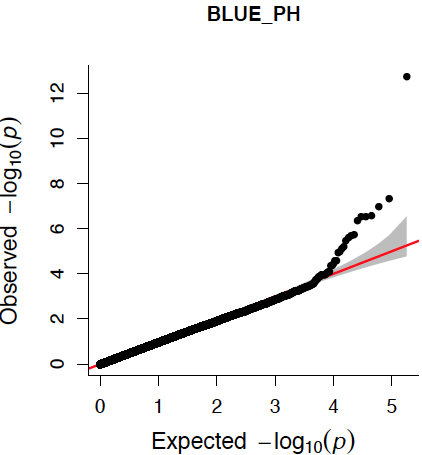


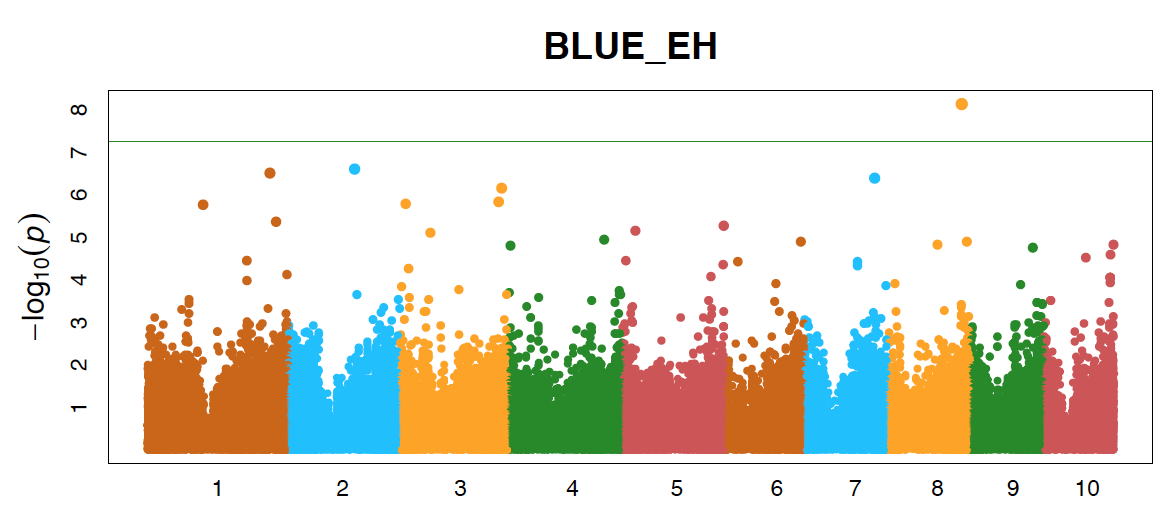

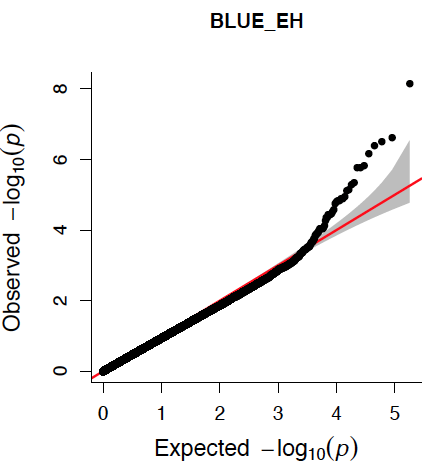

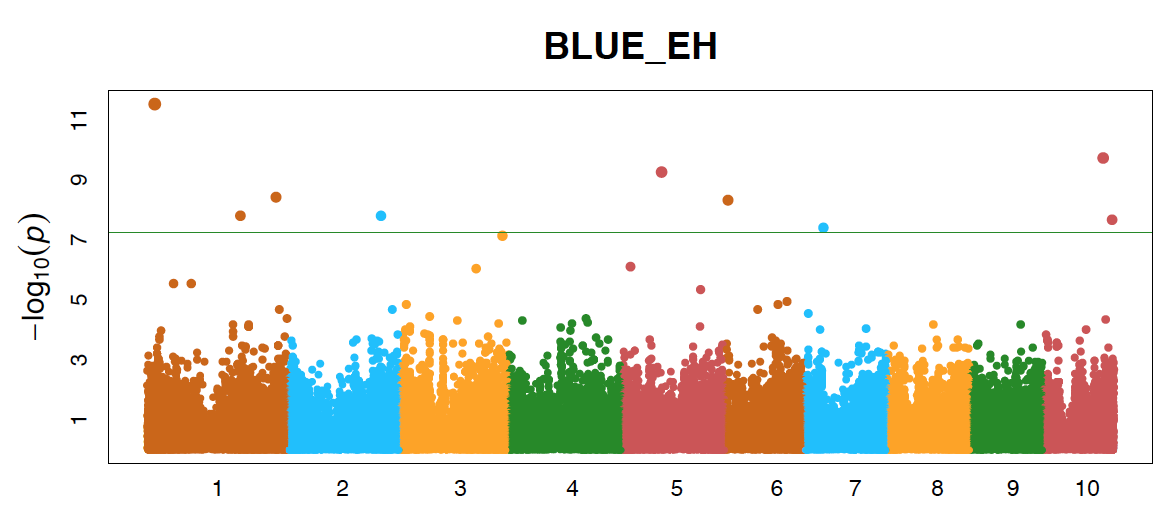

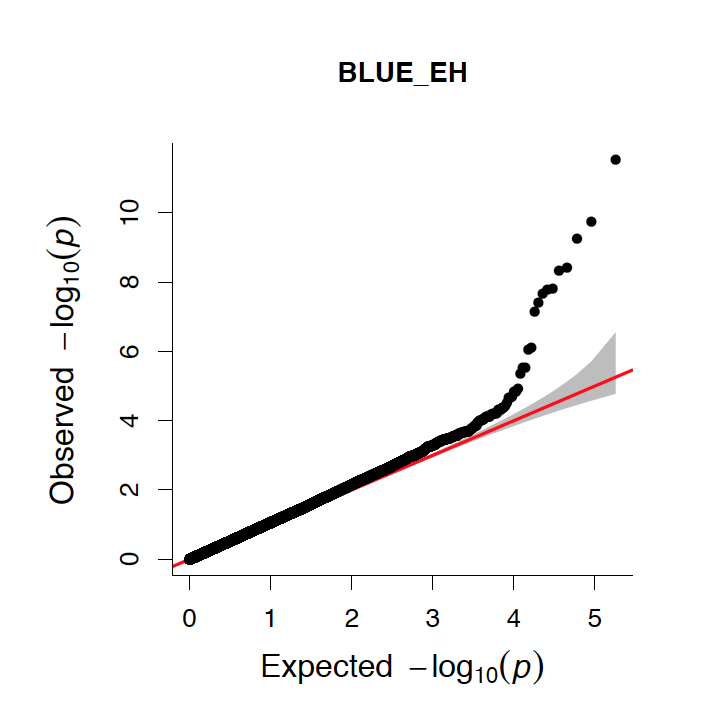


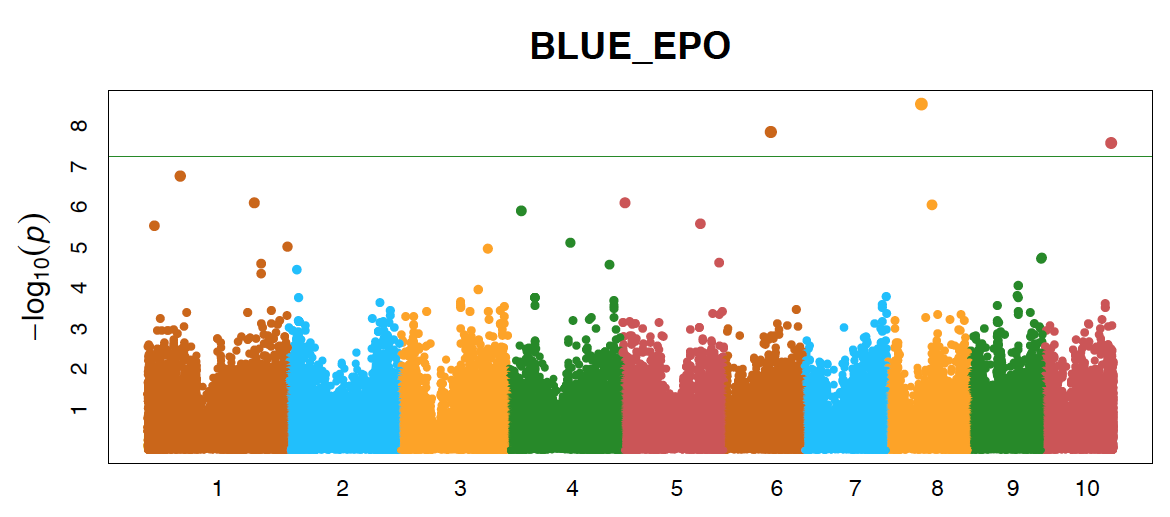

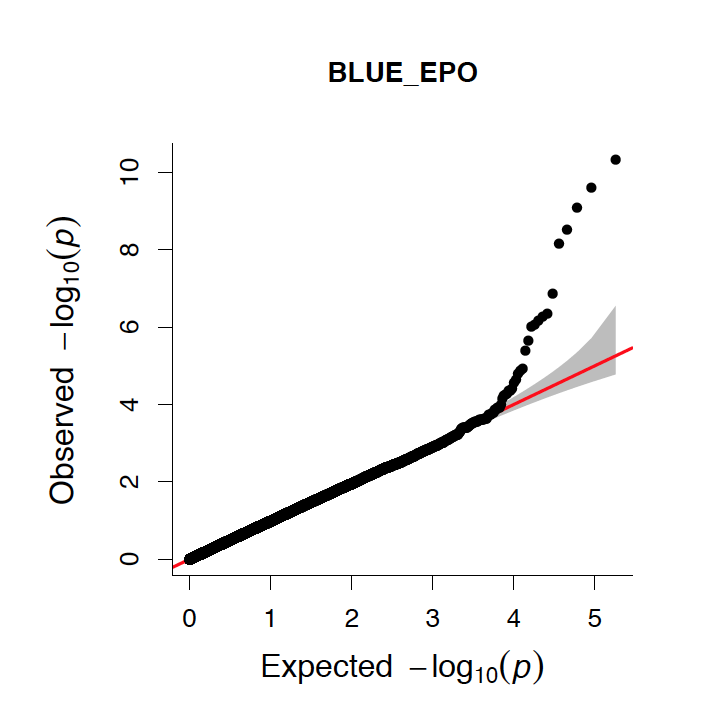

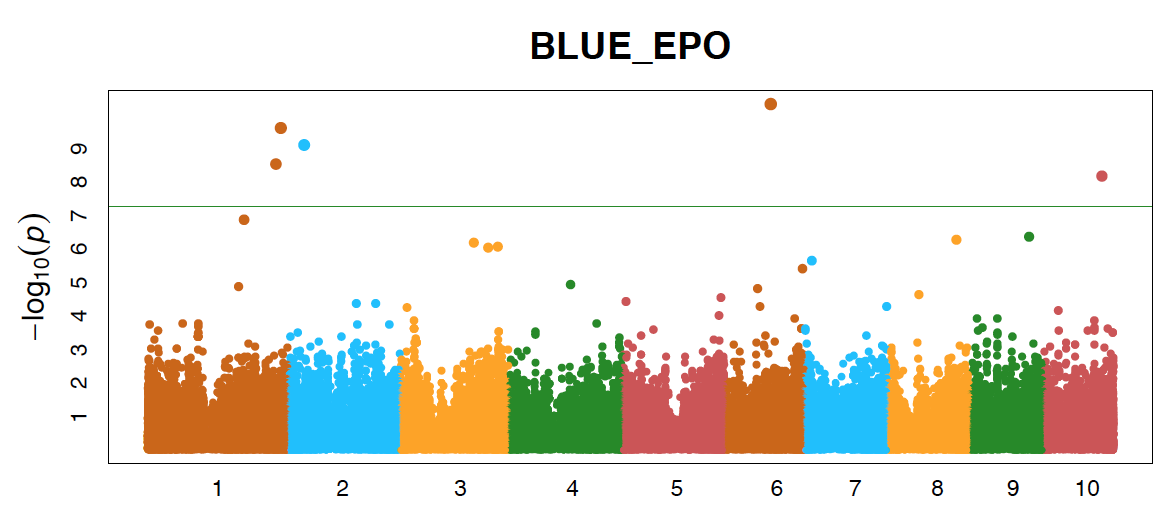

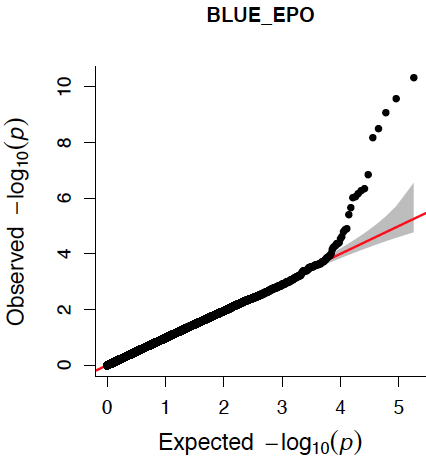


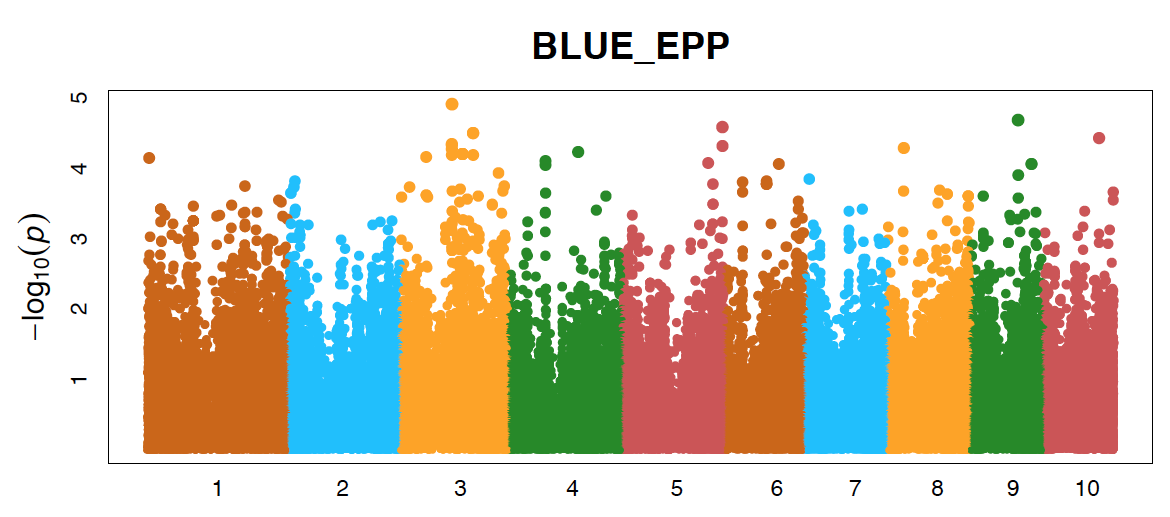

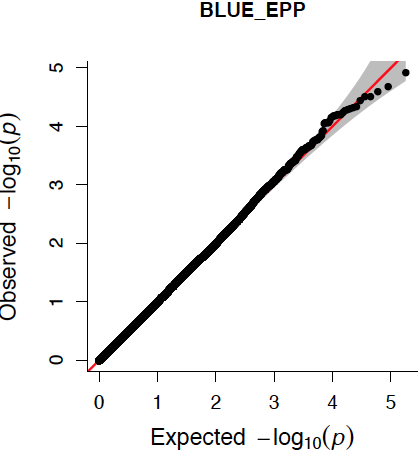

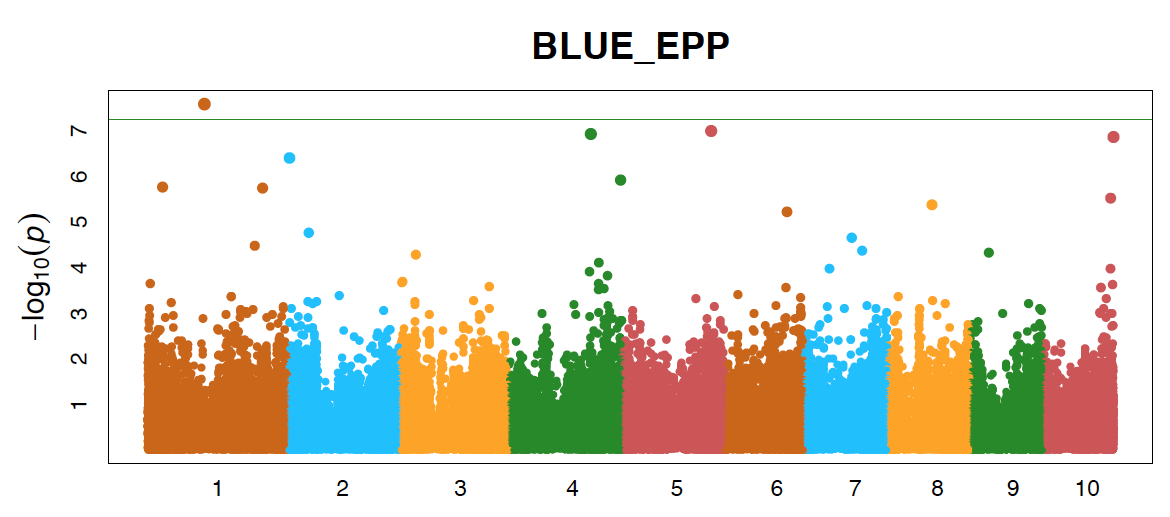

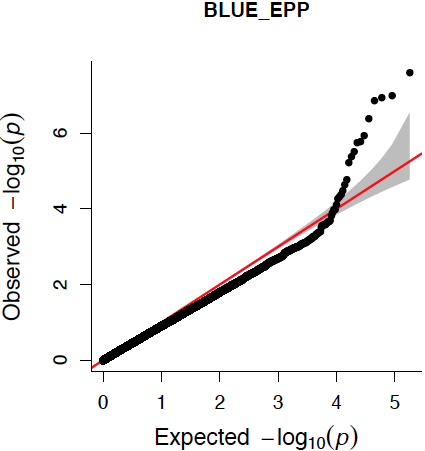


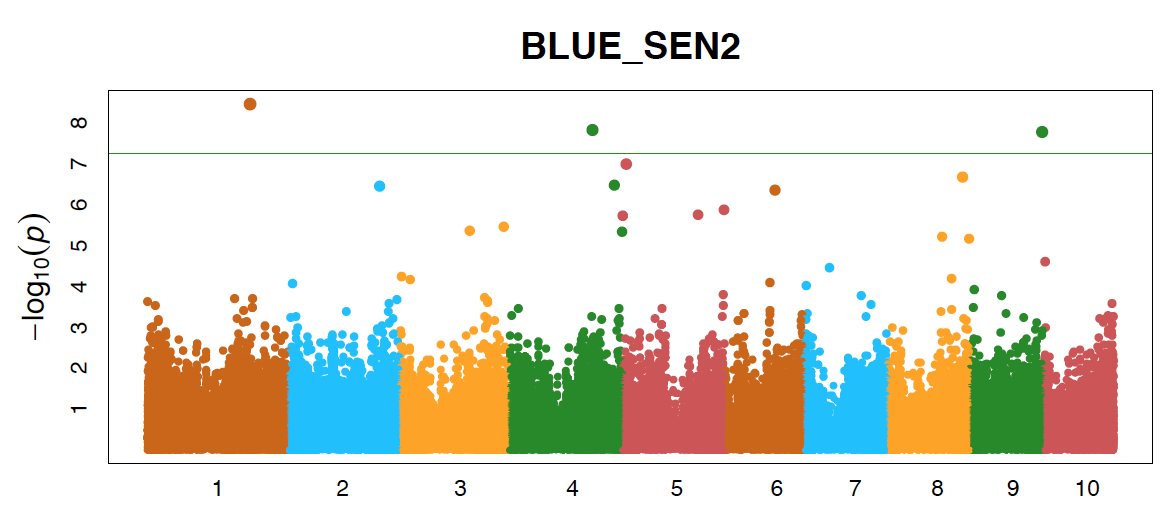

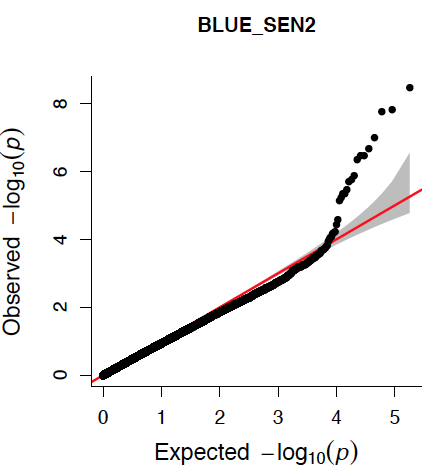

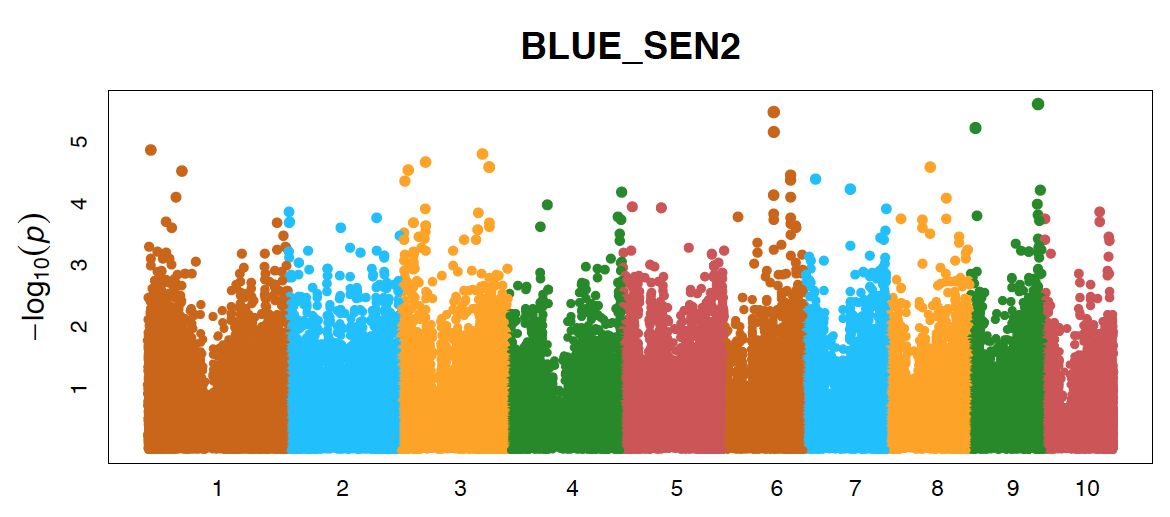

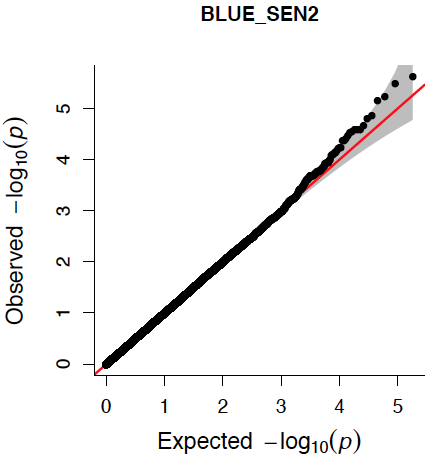


**Supplementary Figure S4.** Manhattan and QQ-plots for GY grain yield, AD days to anthesis, ASI anthesis-silking interval, PH plant height, EH ear height, EPO ear position, EPP ears per plant, and SEN2 senescence under optimum and low N conditions. The horizontal lines at Manhattan plots show the threshold *p* value at Bonferroni cutoff point of 0.05.
