# Supplementary material for: Genetic Dissection of Nitrogen Use Efficiency in Tropical Maize Through Genome-Wide Association and Genomic Prediction
Source: Front Plant Sci. 2020 Apr 28;11:474. doi: 10.3389/fpls.2020.00474 (PMC7198882; doi:10.3389/fpls.2020.00474)
Supplement: Supplementary file 4 [file Table_1.docx]

**Supplementary Table S1.** Putative protein coding genes in linkage disequilibrium with markers significantly associated with eight different traits under optimum and low N conditions.

| **Trait** | **Gene stable ID** | **Gene description** | **Chr** | **Gene Start (bp)** | **Gene End (bp)** | **Gene name** |
| --- | --- | --- | --- | --- | --- | --- |
| GY_Opt | [Zm00001d052069](http://plants.ensembl.org/zea_mays/Gene/Summary?db=core;g=Zm00001d052069) | Putative MYB DNA-binding domain superfamily protein | [4](http://plants.ensembl.org/zea_mays/contigview?chr=4) | [178364585](http://plants.ensembl.org/zea_mays/contigview?chr=4&vc_start=178364585&vc_end=178366789) | [178366789](http://plants.ensembl.org/zea_mays/contigview?chr=4&vc_start=178364585&vc_end=178366789) | - |
| GY_Opt | [Zm00001d017047](http://plants.ensembl.org/zea_mays/Gene/Summary?db=core;g=Zm00001d017047) | 60S ribosomal protein L6 | [5](http://plants.ensembl.org/zea_mays/contigview?chr=5) | [183747380](http://plants.ensembl.org/zea_mays/contigview?chr=5&vc_start=183747380&vc_end=183749370) | [183749370](http://plants.ensembl.org/zea_mays/contigview?chr=5&vc_start=183747380&vc_end=183749370) | - |
| GY_Opt | [Zm00001d009669](http://plants.ensembl.org/zea_mays/Gene/Summary?db=core;g=Zm00001d009669) | Sugar transport1 isoform 1 | [8](http://plants.ensembl.org/zea_mays/contigview?chr=8) | [75146233](http://plants.ensembl.org/zea_mays/contigview?chr=8&vc_start=75146233&vc_end=75152432) | [75152432](http://plants.ensembl.org/zea_mays/contigview?chr=8&vc_start=75146233&vc_end=75152432) | - |
| GY_Opt | [Zm00001d026510](http://plants.ensembl.org/zea_mays/Gene/Summary?db=core;g=Zm00001d026510) | Putative HLH DNA-binding domain superfamily protein | [10](http://plants.ensembl.org/zea_mays/contigview?chr=10) | [147364599](http://plants.ensembl.org/zea_mays/contigview?chr=10&vc_start=147364599&vc_end=147365225) | [147365225](http://plants.ensembl.org/zea_mays/contigview?chr=10&vc_start=147364599&vc_end=147365225) | - |
| GY_Opt | [Zm00001d026514](http://plants.ensembl.org/zea_mays/Gene/Summary?db=core;g=Zm00001d026514) | Inositol 1,3,4,5,6-pentakisphosphate 2-kinase isoform 1 | [10](http://plants.ensembl.org/zea_mays/contigview?chr=10) | [147437436](http://plants.ensembl.org/zea_mays/contigview?chr=10&vc_start=147437436&vc_end=147441371) | [147441371](http://plants.ensembl.org/zea_mays/contigview?chr=10&vc_start=147437436&vc_end=147441371) | - |
| GY_Opt | [Zm00001d026518](http://plants.ensembl.org/zea_mays/Gene/Summary?db=core;g=Zm00001d026518) | BSD domain containing protein | [10](http://plants.ensembl.org/zea_mays/contigview?chr=10) | [147474669](http://plants.ensembl.org/zea_mays/contigview?chr=10&vc_start=147474669&vc_end=147476975) | [147476975](http://plants.ensembl.org/zea_mays/contigview?chr=10&vc_start=147474669&vc_end=147476975) | - |
| GY_Opt | [Zm00001d026521](http://plants.ensembl.org/zea_mays/Gene/Summary?db=core;g=Zm00001d026521) | Inner membrane protease subunit 1 | [10](http://plants.ensembl.org/zea_mays/contigview?chr=10) | [147481845](http://plants.ensembl.org/zea_mays/contigview?chr=10&vc_start=147481845&vc_end=147487342) | [147487342](http://plants.ensembl.org/zea_mays/contigview?chr=10&vc_start=147481845&vc_end=147487342) | - |
| GY_Opt | [Zm00001d026537](http://plants.ensembl.org/zea_mays/Gene/Summary?db=core;g=Zm00001d026537) | Putative homeobox DNA-binding domain superfamily protein | [10](http://plants.ensembl.org/zea_mays/contigview?chr=10) | [147855536](http://plants.ensembl.org/zea_mays/contigview?chr=10&vc_start=147855536&vc_end=147856873) | [147856873](http://plants.ensembl.org/zea_mays/contigview?chr=10&vc_start=147855536&vc_end=147856873) | - |
| GY_LowN | [Zm00001d031808](http://plants.ensembl.org/zea_mays/Gene/Summary?db=core;g=Zm00001d031808) | Short-chain dehydrogenase/reductase SDR | [1](http://plants.ensembl.org/zea_mays/contigview?chr=1) | [202464156](http://plants.ensembl.org/zea_mays/contigview?chr=1&vc_start=202464156&vc_end=202467819) | [202467819](http://plants.ensembl.org/zea_mays/contigview?chr=1&vc_start=202464156&vc_end=202467819) | - |
| GY_LowN | [Zm00001d031811](http://plants.ensembl.org/zea_mays/Gene/Summary?db=core;g=Zm00001d031811) | Patatin | [1](http://plants.ensembl.org/zea_mays/contigview?chr=1) | [202554373](http://plants.ensembl.org/zea_mays/contigview?chr=1&vc_start=202554373&vc_end=202556086) | [202556086](http://plants.ensembl.org/zea_mays/contigview?chr=1&vc_start=202554373&vc_end=202556086) | - |
| GY_LowN | [Zm00001d016248](http://plants.ensembl.org/zea_mays/Gene/Summary?db=core;g=Zm00001d016248) | Citrate synthase | [5](http://plants.ensembl.org/zea_mays/contigview?chr=5) | [152956416](http://plants.ensembl.org/zea_mays/contigview?chr=5&vc_start=152956416&vc_end=152961046) | [152961046](http://plants.ensembl.org/zea_mays/contigview?chr=5&vc_start=152956416&vc_end=152961046) | - |
| GY_LowN | [Zm00001d018089](http://plants.ensembl.org/zea_mays/Gene/Summary?db=core;g=Zm00001d018089) | VQ motif family protein | [5](http://plants.ensembl.org/zea_mays/contigview?chr=5) | [214041553](http://plants.ensembl.org/zea_mays/contigview?chr=5&vc_start=214041553&vc_end=214042179) | [214042179](http://plants.ensembl.org/zea_mays/contigview?chr=5&vc_start=214041553&vc_end=214042179) | - |
| GY_LowN | [Zm00001d018090](http://plants.ensembl.org/zea_mays/Gene/Summary?db=core;g=Zm00001d018090) | Annexin | [5](http://plants.ensembl.org/zea_mays/contigview?chr=5) | [214042874](http://plants.ensembl.org/zea_mays/contigview?chr=5&vc_start=214042874&vc_end=214045954) | [214045954](http://plants.ensembl.org/zea_mays/contigview?chr=5&vc_start=214042874&vc_end=214045954) | - |
| GY_LowN | [Zm00001d018099](http://plants.ensembl.org/zea_mays/Gene/Summary?db=core;g=Zm00001d018099) | Adenosine 5'-phosphosulfate reductase 4 | [5](http://plants.ensembl.org/zea_mays/contigview?chr=5) | [214157648](http://plants.ensembl.org/zea_mays/contigview?chr=5&vc_start=214157648&vc_end=214160124) | [214160124](http://plants.ensembl.org/zea_mays/contigview?chr=5&vc_start=214157648&vc_end=214160124) | - |
| GY_LowN | [Zm00001d020692](http://plants.ensembl.org/zea_mays/Gene/Summary?db=core;g=Zm00001d020692) | Hexosyltransferase | [7](http://plants.ensembl.org/zea_mays/contigview?chr=7) | [128747473](http://plants.ensembl.org/zea_mays/contigview?chr=7&vc_start=128747473&vc_end=128750503) | [128750503](http://plants.ensembl.org/zea_mays/contigview?chr=7&vc_start=128747473&vc_end=128750503) | - |
| GY_LowN | [Zm00001d020693](http://plants.ensembl.org/zea_mays/Gene/Summary?db=core;g=Zm00001d020693) | Putative uncharacterized protein | [7](http://plants.ensembl.org/zea_mays/contigview?chr=7) | [128776562](http://plants.ensembl.org/zea_mays/contigview?chr=7&vc_start=128776562&vc_end=128777569) | [128777569](http://plants.ensembl.org/zea_mays/contigview?chr=7&vc_start=128776562&vc_end=128777569) | - |
| AD_Opt | [Zm00001d021569](http://plants.ensembl.org/zea_mays/Gene/Summary?db=core;g=Zm00001d021569) | Protein DETOXIFICATION | [7](http://plants.ensembl.org/zea_mays/contigview?chr=7) | [156294946](http://plants.ensembl.org/zea_mays/contigview?chr=7&vc_start=156294946&vc_end=156300431) | [156300431](http://plants.ensembl.org/zea_mays/contigview?chr=7&vc_start=156294946&vc_end=156300431) | - |
| AD_Opt | [Zm00001d021576](http://plants.ensembl.org/zea_mays/Gene/Summary?db=core;g=Zm00001d021576) | Glycosyltransferase | [7](http://plants.ensembl.org/zea_mays/contigview?chr=7) | [156641990](http://plants.ensembl.org/zea_mays/contigview?chr=7&vc_start=156641990&vc_end=156643471) | [156643471](http://plants.ensembl.org/zea_mays/contigview?chr=7&vc_start=156641990&vc_end=156643471) | - |
| AD_Opt | [Zm00001d012220](http://plants.ensembl.org/zea_mays/Gene/Summary?db=core;g=Zm00001d012220) | Putative ENTH/ANTH/VHS superfamily protein | [8](http://plants.ensembl.org/zea_mays/contigview?chr=8) | [170362957](http://plants.ensembl.org/zea_mays/contigview?chr=8&vc_start=170362957&vc_end=170364624) | [170364624](http://plants.ensembl.org/zea_mays/contigview?chr=8&vc_start=170362957&vc_end=170364624) | - |
| AD_Opt | [Zm00001d012221](http://plants.ensembl.org/zea_mays/Gene/Summary?db=core;g=Zm00001d012221) | Acyl-desaturase | [8](http://plants.ensembl.org/zea_mays/contigview?chr=8) | [170366926](http://plants.ensembl.org/zea_mays/contigview?chr=8&vc_start=170366926&vc_end=170368457) | [170368457](http://plants.ensembl.org/zea_mays/contigview?chr=8&vc_start=170366926&vc_end=170368457) | - |
| AD_Opt | [Zm00001d012224](http://plants.ensembl.org/zea_mays/Gene/Summary?db=core;g=Zm00001d012224) | Hexosyltransferase | [8](http://plants.ensembl.org/zea_mays/contigview?chr=8) | [170419466](http://plants.ensembl.org/zea_mays/contigview?chr=8&vc_start=170419466&vc_end=170424898) | [170424898](http://plants.ensembl.org/zea_mays/contigview?chr=8&vc_start=170419466&vc_end=170424898) | - |
| AD_Opt | [Zm00001d012228](http://plants.ensembl.org/zea_mays/Gene/Summary?db=core;g=Zm00001d012228) | 4,5-DOPA dioxygenase extradiol | [8](http://plants.ensembl.org/zea_mays/contigview?chr=8) | [170458719](http://plants.ensembl.org/zea_mays/contigview?chr=8&vc_start=170458719&vc_end=170462003) | [170462003](http://plants.ensembl.org/zea_mays/contigview?chr=8&vc_start=170458719&vc_end=170462003) | - |
| AD_LowN | [Zm00001d033822](http://plants.ensembl.org/zea_mays/Gene/Summary?db=core;g=Zm00001d033822) | Histone H2A | [1](http://plants.ensembl.org/zea_mays/contigview?chr=1) | [274809375](http://plants.ensembl.org/zea_mays/contigview?chr=1&vc_start=274809375&vc_end=274810292) | [274810292](http://plants.ensembl.org/zea_mays/contigview?chr=1&vc_start=274809375&vc_end=274810292) | - |
| AD_LowN | [Zm00001d033823](http://plants.ensembl.org/zea_mays/Gene/Summary?db=core;g=Zm00001d033823) | Putative uncharacterized protein | [1](http://plants.ensembl.org/zea_mays/contigview?chr=1) | [274819675](http://plants.ensembl.org/zea_mays/contigview?chr=1&vc_start=274819675&vc_end=274820688) | [274820688](http://plants.ensembl.org/zea_mays/contigview?chr=1&vc_start=274819675&vc_end=274820688) | - |
| AD_LowN | [Zm00001d033830](http://plants.ensembl.org/zea_mays/Gene/Summary?db=core;g=Zm00001d033830) | Adenylyl cyclase-associated protein | [1](http://plants.ensembl.org/zea_mays/contigview?chr=1) | [275160006](http://plants.ensembl.org/zea_mays/contigview?chr=1&vc_start=275160006&vc_end=275165400) | [275165400](http://plants.ensembl.org/zea_mays/contigview?chr=1&vc_start=275160006&vc_end=275165400) | - |
| AD_LowN | [Zm00001d004689](http://plants.ensembl.org/zea_mays/Gene/Summary?db=core;g=Zm00001d004689) | Herbicide safener binding protein | [2](http://plants.ensembl.org/zea_mays/contigview?chr=2) | [131242393](http://plants.ensembl.org/zea_mays/contigview?chr=2&vc_start=131242393&vc_end=131243911) | [131243911](http://plants.ensembl.org/zea_mays/contigview?chr=2&vc_start=131242393&vc_end=131243911) | - |
| AD_LowN | [Zm00001d038186](http://plants.ensembl.org/zea_mays/Gene/Summary?db=core;g=Zm00001d038186) | Peptide transporter PTR2 | [6](http://plants.ensembl.org/zea_mays/contigview?chr=6) | [150861505](http://plants.ensembl.org/zea_mays/contigview?chr=6&vc_start=150861505&vc_end=150866590) | [150866590](http://plants.ensembl.org/zea_mays/contigview?chr=6&vc_start=150861505&vc_end=150866590) | - |
| AD_LowN | [Zm00001d020580](http://plants.ensembl.org/zea_mays/Gene/Summary?db=core;g=Zm00001d020580) | Histone H2B | [7](http://plants.ensembl.org/zea_mays/contigview?chr=7) | [123664001](http://plants.ensembl.org/zea_mays/contigview?chr=7&vc_start=123664001&vc_end=123664456) | [123664456](http://plants.ensembl.org/zea_mays/contigview?chr=7&vc_start=123664001&vc_end=123664456) | - |
| AD_LowN | [Zm00001d020583](http://plants.ensembl.org/zea_mays/Gene/Summary?db=core;g=Zm00001d020583) | Hexosyltransferase | [7](http://plants.ensembl.org/zea_mays/contigview?chr=7) | [123675725](http://plants.ensembl.org/zea_mays/contigview?chr=7&vc_start=123675725&vc_end=123676681) | [123676681](http://plants.ensembl.org/zea_mays/contigview?chr=7&vc_start=123675725&vc_end=123676681) | - |
| AD_LowN | [Zm00001d020584](http://plants.ensembl.org/zea_mays/Gene/Summary?db=core;g=Zm00001d020584) | Histone H4 | [7](http://plants.ensembl.org/zea_mays/contigview?chr=7) | [123703834](http://plants.ensembl.org/zea_mays/contigview?chr=7&vc_start=123703834&vc_end=123704145) | [123704145](http://plants.ensembl.org/zea_mays/contigview?chr=7&vc_start=123703834&vc_end=123704145) | [H4C7](http://plants.ensembl.org/zea_mays/Gene/Summary?db=core;g=Zm00001d020584) |
| AD_LowN | [Zm00001d020586](http://plants.ensembl.org/zea_mays/Gene/Summary?db=core;g=Zm00001d020586) | Pectinesterase | [7](http://plants.ensembl.org/zea_mays/contigview?chr=7) | [123729032](http://plants.ensembl.org/zea_mays/contigview?chr=7&vc_start=123729032&vc_end=123731282) | [123731282](http://plants.ensembl.org/zea_mays/contigview?chr=7&vc_start=123729032&vc_end=123731282) | - |
| AD_LowN | [Zm00001d020591](http://plants.ensembl.org/zea_mays/Gene/Summary?db=core;g=Zm00001d020591) | 50 kDa gamma-zein | [7](http://plants.ensembl.org/zea_mays/contigview?chr=7) | [123954263](http://plants.ensembl.org/zea_mays/contigview?chr=7&vc_start=123954263&vc_end=123955189) | [123955189](http://plants.ensembl.org/zea_mays/contigview?chr=7&vc_start=123954263&vc_end=123955189) | - |
| AD_LowN | [Zm00001d020592](http://plants.ensembl.org/zea_mays/Gene/Summary?db=core;g=Zm00001d020592) | 27 kDa gamma-zein | [7](http://plants.ensembl.org/zea_mays/contigview?chr=7) | [123982344](http://plants.ensembl.org/zea_mays/contigview?chr=7&vc_start=123982344&vc_end=123983015) | [123983015](http://plants.ensembl.org/zea_mays/contigview?chr=7&vc_start=123982344&vc_end=123983015) | - |
| AD_LowN | [Zm00001d025706](http://plants.ensembl.org/zea_mays/Gene/Summary?db=core;g=Zm00001d025706) | CFM6 | [10](http://plants.ensembl.org/zea_mays/contigview?chr=10) | [126880549](http://plants.ensembl.org/zea_mays/contigview?chr=10&vc_start=126880549&vc_end=126888843) | [126888843](http://plants.ensembl.org/zea_mays/contigview?chr=10&vc_start=126880549&vc_end=126888843) | - |
| AD_LowN | [Zm00001d026540](http://plants.ensembl.org/zea_mays/Gene/Summary?db=core;g=Zm00001d026540) | Auxin response factor | [10](http://plants.ensembl.org/zea_mays/contigview?chr=10) | [147919136](http://plants.ensembl.org/zea_mays/contigview?chr=10&vc_start=147919136&vc_end=147925555) | [147925555](http://plants.ensembl.org/zea_mays/contigview?chr=10&vc_start=147919136&vc_end=147925555) | - |
| AD_LowN | [Zm00001d026542](http://plants.ensembl.org/zea_mays/Gene/Summary?db=core;g=Zm00001d026542) | G2-like transcription factor | [10](http://plants.ensembl.org/zea_mays/contigview?chr=10) | [147942412](http://plants.ensembl.org/zea_mays/contigview?chr=10&vc_start=147942412&vc_end=147945498) | [147945498](http://plants.ensembl.org/zea_mays/contigview?chr=10&vc_start=147942412&vc_end=147945498) | - |
| AD_LowN | [Zm00001d026546](http://plants.ensembl.org/zea_mays/Gene/Summary?db=core;g=Zm00001d026546) | Dirigent protein | [10](http://plants.ensembl.org/zea_mays/contigview?chr=10) | [147981376](http://plants.ensembl.org/zea_mays/contigview?chr=10&vc_start=147981376&vc_end=147981964) | [147981964](http://plants.ensembl.org/zea_mays/contigview?chr=10&vc_start=147981376&vc_end=147981964) | - |
| AD_LowN | [Zm00001d026547](http://plants.ensembl.org/zea_mays/Gene/Summary?db=core;g=Zm00001d026547) | Trafficking protein particle complex subunit 3 | [10](http://plants.ensembl.org/zea_mays/contigview?chr=10) | [147985161](http://plants.ensembl.org/zea_mays/contigview?chr=10&vc_start=147985161&vc_end=147988097) | [147988097](http://plants.ensembl.org/zea_mays/contigview?chr=10&vc_start=147985161&vc_end=147988097) | - |
| AD_LowN | [Zm00001d006539](http://plants.ensembl.org/zea_mays/Gene/Summary?db=core;g=Zm00001d006539) | Beta-amylase | [2](http://plants.ensembl.org/zea_mays/contigview?chr=2) | [210700847](http://plants.ensembl.org/zea_mays/contigview?chr=2&vc_start=210700847&vc_end=210703733) | [210703733](http://plants.ensembl.org/zea_mays/contigview?chr=2&vc_start=210700847&vc_end=210703733) | - |
| ASI_Opt | [Zm00001d002149](http://plants.ensembl.org/zea_mays/Gene/Summary?db=core;g=Zm00001d002149) | Hexosyltransferase | [2](http://plants.ensembl.org/zea_mays/contigview?chr=2) | [6819751](http://plants.ensembl.org/zea_mays/contigview?chr=2&vc_start=6819751&vc_end=6827540) | [6827540](http://plants.ensembl.org/zea_mays/contigview?chr=2&vc_start=6819751&vc_end=6827540) | - |
| ASI_Opt | [Zm00001d053580](http://plants.ensembl.org/zea_mays/Gene/Summary?db=core;g=Zm00001d053580) | Acetyltransferase component of pyruvate dehydrogenase complex | [4](http://plants.ensembl.org/zea_mays/contigview?chr=4) | [235225983](http://plants.ensembl.org/zea_mays/contigview?chr=4&vc_start=235225983&vc_end=235235156) | [235235156](http://plants.ensembl.org/zea_mays/contigview?chr=4&vc_start=235225983&vc_end=235235156) | - |
| ASI_Opt | [Zm00001d019262](http://plants.ensembl.org/zea_mays/Gene/Summary?db=core;g=Zm00001d019262) | Proteasome subunit beta type | [7](http://plants.ensembl.org/zea_mays/contigview?chr=7) | [24655394](http://plants.ensembl.org/zea_mays/contigview?chr=7&vc_start=24655394&vc_end=24659033) | [24659033](http://plants.ensembl.org/zea_mays/contigview?chr=7&vc_start=24655394&vc_end=24659033) | - |
| ASI_Opt | [Zm00001d021544](http://plants.ensembl.org/zea_mays/Gene/Summary?db=core;g=Zm00001d021544) | PHI-1 | [7](http://plants.ensembl.org/zea_mays/contigview?chr=7) | [155503583](http://plants.ensembl.org/zea_mays/contigview?chr=7&vc_start=155503583&vc_end=155504602) | [155504602](http://plants.ensembl.org/zea_mays/contigview?chr=7&vc_start=155503583&vc_end=155504602) | - |
| ASI_Opt | [Zm00001d021546](http://plants.ensembl.org/zea_mays/Gene/Summary?db=core;g=Zm00001d021546) | Malic enzyme | [7](http://plants.ensembl.org/zea_mays/contigview?chr=7) | [155518816](http://plants.ensembl.org/zea_mays/contigview?chr=7&vc_start=155518816&vc_end=155528949) | [155528949](http://plants.ensembl.org/zea_mays/contigview?chr=7&vc_start=155518816&vc_end=155528949) | - |
| ASI_Opt | [Zm00001d021554](http://plants.ensembl.org/zea_mays/Gene/Summary?db=core;g=Zm00001d021554) | ATP synthase delta chain | [7](http://plants.ensembl.org/zea_mays/contigview?chr=7) | [155779197](http://plants.ensembl.org/zea_mays/contigview?chr=7&vc_start=155779197&vc_end=155787322) | [155787322](http://plants.ensembl.org/zea_mays/contigview?chr=7&vc_start=155779197&vc_end=155787322) | - |
| ASI_Opt | [Zm00001d010985](http://plants.ensembl.org/zea_mays/Gene/Summary?db=core;g=Zm00001d010985) | Organic anion transporter | [8](http://plants.ensembl.org/zea_mays/contigview?chr=8) | [135928640](http://plants.ensembl.org/zea_mays/contigview?chr=8&vc_start=135928640&vc_end=135930519) | [135930519](http://plants.ensembl.org/zea_mays/contigview?chr=8&vc_start=135928640&vc_end=135930519) | - |
| ASI_Opt | [Zm00001d010994](http://plants.ensembl.org/zea_mays/Gene/Summary?db=core;g=Zm00001d010994) | Structural constituent of ribosome | [8](http://plants.ensembl.org/zea_mays/contigview?chr=8) | [136140894](http://plants.ensembl.org/zea_mays/contigview?chr=8&vc_start=136140894&vc_end=136141484) | [136141484](http://plants.ensembl.org/zea_mays/contigview?chr=8&vc_start=136140894&vc_end=136141484) | - |
| ASI_Opt | [Zm00001d010998](http://plants.ensembl.org/zea_mays/Gene/Summary?db=core;g=Zm00001d010998) | Putative homeobox DNA-binding domain superfamily protein | [8](http://plants.ensembl.org/zea_mays/contigview?chr=8) | [136302778](http://plants.ensembl.org/zea_mays/contigview?chr=8&vc_start=136302778&vc_end=136306209) | [136306209](http://plants.ensembl.org/zea_mays/contigview?chr=8&vc_start=136302778&vc_end=136306209) | - |
| ASI_Opt | [Zm00001d011001](http://plants.ensembl.org/zea_mays/Gene/Summary?db=core;g=Zm00001d011001) | Membrane protein | [8](http://plants.ensembl.org/zea_mays/contigview?chr=8) | [136349224](http://plants.ensembl.org/zea_mays/contigview?chr=8&vc_start=136349224&vc_end=136350587) | [136350587](http://plants.ensembl.org/zea_mays/contigview?chr=8&vc_start=136349224&vc_end=136350587) | - |
| ASI_Opt | [Zm00001d047081](http://plants.ensembl.org/zea_mays/Gene/Summary?db=core;g=Zm00001d047081) | C2C2-GATA transcription factor | [9](http://plants.ensembl.org/zea_mays/contigview?chr=9) | [117752835](http://plants.ensembl.org/zea_mays/contigview?chr=9&vc_start=117752835&vc_end=117754586) | [117754586](http://plants.ensembl.org/zea_mays/contigview?chr=9&vc_start=117752835&vc_end=117754586) | - |
| ASI_Opt | [Zm00001d047087](http://plants.ensembl.org/zea_mays/Gene/Summary?db=core;g=Zm00001d047087) | Beta-expansin 1a | [9](http://plants.ensembl.org/zea_mays/contigview?chr=9) | [118041045](http://plants.ensembl.org/zea_mays/contigview?chr=9&vc_start=118041045&vc_end=118043785) | [118043785](http://plants.ensembl.org/zea_mays/contigview?chr=9&vc_start=118041045&vc_end=118043785) | - |
| ASI_Opt | [Zm00001d047089](http://plants.ensembl.org/zea_mays/Gene/Summary?db=core;g=Zm00001d047089) | Beta-expansin 5 | [9](http://plants.ensembl.org/zea_mays/contigview?chr=9) | [118067914](http://plants.ensembl.org/zea_mays/contigview?chr=9&vc_start=118067914&vc_end=118069999) | [118069999](http://plants.ensembl.org/zea_mays/contigview?chr=9&vc_start=118067914&vc_end=118069999) | - |
| ASI_LowN | [Zm00001d027453](http://plants.ensembl.org/zea_mays/Gene/Summary?db=core;g=Zm00001d027453) | Plant-specific domain TIGR01568 family protein | [1](http://plants.ensembl.org/zea_mays/contigview?chr=1) | [5589770](http://plants.ensembl.org/zea_mays/contigview?chr=1&vc_start=5589770&vc_end=5590513) | [5590513](http://plants.ensembl.org/zea_mays/contigview?chr=1&vc_start=5589770&vc_end=5590513) | - |
| ASI_LowN | [Zm00001d027455](http://plants.ensembl.org/zea_mays/Gene/Summary?db=core;g=Zm00001d027455) | Non-specific serine/threonine protein kinase | [1](http://plants.ensembl.org/zea_mays/contigview?chr=1) | [5610415](http://plants.ensembl.org/zea_mays/contigview?chr=1&vc_start=5610415&vc_end=5614949) | [5614949](http://plants.ensembl.org/zea_mays/contigview?chr=1&vc_start=5610415&vc_end=5614949) | - |
| ASI_LowN | [Zm00001d027456](http://plants.ensembl.org/zea_mays/Gene/Summary?db=core;g=Zm00001d027456) | Threonine endopeptidase | [1](http://plants.ensembl.org/zea_mays/contigview?chr=1) | [5661737](http://plants.ensembl.org/zea_mays/contigview?chr=1&vc_start=5661737&vc_end=5663089) | [5663089](http://plants.ensembl.org/zea_mays/contigview?chr=1&vc_start=5661737&vc_end=5663089) | - |
| ASI_LowN | [Zm00001d027458](http://plants.ensembl.org/zea_mays/Gene/Summary?db=core;g=Zm00001d027458) | Cell growth defect factor 2 | [1](http://plants.ensembl.org/zea_mays/contigview?chr=1) | [5701680](http://plants.ensembl.org/zea_mays/contigview?chr=1&vc_start=5701680&vc_end=5704055) | [5704055](http://plants.ensembl.org/zea_mays/contigview?chr=1&vc_start=5701680&vc_end=5704055) | - |
| ASI_LowN | [Zm00001d027468](http://plants.ensembl.org/zea_mays/Gene/Summary?db=core;g=Zm00001d027468) | ADP,ATP carrier protein | [1](http://plants.ensembl.org/zea_mays/contigview?chr=1) | [5943755](http://plants.ensembl.org/zea_mays/contigview?chr=1&vc_start=5943755&vc_end=5947624) | [5947624](http://plants.ensembl.org/zea_mays/contigview?chr=1&vc_start=5943755&vc_end=5947624) | - |
| ASI_LowN | [Zm00001d027469](http://plants.ensembl.org/zea_mays/Gene/Summary?db=core;g=Zm00001d027469) | Cytochrome c oxidase assembly protein COX19 | [1](http://plants.ensembl.org/zea_mays/contigview?chr=1) | [6020779](http://plants.ensembl.org/zea_mays/contigview?chr=1&vc_start=6020779&vc_end=6023318) | [6023318](http://plants.ensembl.org/zea_mays/contigview?chr=1&vc_start=6020779&vc_end=6023318) | - |
| ASI_LowN | [Zm00001d007267](http://plants.ensembl.org/zea_mays/Gene/Summary?db=core;g=Zm00001d007267) | Chlorophyll a-b binding protein, chloroplastic | [2](http://plants.ensembl.org/zea_mays/contigview?chr=2) | [226141607](http://plants.ensembl.org/zea_mays/contigview?chr=2&vc_start=226141607&vc_end=226143698) | [226143698](http://plants.ensembl.org/zea_mays/contigview?chr=2&vc_start=226141607&vc_end=226143698) | - |
| ASI_LowN | [Zm00001d042019](http://plants.ensembl.org/zea_mays/Gene/Summary?db=core;g=Zm00001d042019) | Peroxidase | [3](http://plants.ensembl.org/zea_mays/contigview?chr=3) | [147549893](http://plants.ensembl.org/zea_mays/contigview?chr=3&vc_start=147549893&vc_end=147551603) | [147551603](http://plants.ensembl.org/zea_mays/contigview?chr=3&vc_start=147549893&vc_end=147551603) | - |
| ASI_LowN | [Zm00001d042013](http://plants.ensembl.org/zea_mays/Gene/Summary?db=core;g=Zm00001d042013) | Serine/threonine-protein kinase | [3](http://plants.ensembl.org/zea_mays/contigview?chr=3) | [147399992](http://plants.ensembl.org/zea_mays/contigview?chr=3&vc_start=147399992&vc_end=147402361) | [147402361](http://plants.ensembl.org/zea_mays/contigview?chr=3&vc_start=147399992&vc_end=147402361) | - |
| ASI_LowN | [Zm00001d049608](http://plants.ensembl.org/zea_mays/Gene/Summary?db=core;g=Zm00001d049608) | FIE1 | [4](http://plants.ensembl.org/zea_mays/contigview?chr=4) | [37421922](http://plants.ensembl.org/zea_mays/contigview?chr=4&vc_start=37421922&vc_end=37427789) | [37427789](http://plants.ensembl.org/zea_mays/contigview?chr=4&vc_start=37421922&vc_end=37427789) | [FIE1](http://plants.ensembl.org/zea_mays/Gene/Summary?db=core;g=Zm00001d049608) |
| ASI_LowN | [Zm00001d038784](http://plants.ensembl.org/zea_mays/Gene/Summary?db=core;g=Zm00001d038784) | Auxin-responsive protein | [6](http://plants.ensembl.org/zea_mays/contigview?chr=6) | [164332917](http://plants.ensembl.org/zea_mays/contigview?chr=6&vc_start=164332917&vc_end=164336272) | [164336272](http://plants.ensembl.org/zea_mays/contigview?chr=6&vc_start=164332917&vc_end=164336272) | - |
| ASI_LowN | [Zm00001d038792](http://plants.ensembl.org/zea_mays/Gene/Summary?db=core;g=Zm00001d038792) | Phosphotransferase | [6](http://plants.ensembl.org/zea_mays/contigview?chr=6) | [164389095](http://plants.ensembl.org/zea_mays/contigview?chr=6&vc_start=164389095&vc_end=164395441) | [164395441](http://plants.ensembl.org/zea_mays/contigview?chr=6&vc_start=164389095&vc_end=164395441) | - |
| ASI_LowN | [Zm00001d038794](http://plants.ensembl.org/zea_mays/Gene/Summary?db=core;g=Zm00001d038794) | Pectinesterase | [6](http://plants.ensembl.org/zea_mays/contigview?chr=6) | [164493063](http://plants.ensembl.org/zea_mays/contigview?chr=6&vc_start=164493063&vc_end=164495421) | [164495421](http://plants.ensembl.org/zea_mays/contigview?chr=6&vc_start=164493063&vc_end=164495421) | - |
| ASI_LowN | [Zm00001d023395](http://plants.ensembl.org/zea_mays/Gene/Summary?db=core;g=Zm00001d023395) | Pop3 peptide | [10](http://plants.ensembl.org/zea_mays/contigview?chr=10) | [4672326](http://plants.ensembl.org/zea_mays/contigview?chr=10&vc_start=4672326&vc_end=4673327) | [4673327](http://plants.ensembl.org/zea_mays/contigview?chr=10&vc_start=4672326&vc_end=4673327) | - |
| ASI_LowN | [Zm00001d023396](http://plants.ensembl.org/zea_mays/Gene/Summary?db=core;g=Zm00001d023396) | Putative RING zinc finger domain superfamily protein | [10](http://plants.ensembl.org/zea_mays/contigview?chr=10) | [4674375](http://plants.ensembl.org/zea_mays/contigview?chr=10&vc_start=4674375&vc_end=4678089) | [4678089](http://plants.ensembl.org/zea_mays/contigview?chr=10&vc_start=4674375&vc_end=4678089) | - |
| EH_Opt | [Zm00001d004807](http://plants.ensembl.org/zea_mays/Gene/Summary?db=core;g=Zm00001d004807) | Coatomer subunit beta | [2](http://plants.ensembl.org/zea_mays/contigview?chr=2) | [140587723](http://plants.ensembl.org/zea_mays/contigview?chr=2&vc_start=140587723&vc_end=140592773) | [140592773](http://plants.ensembl.org/zea_mays/contigview?chr=2&vc_start=140587723&vc_end=140592773) | - |
| EH_Opt | [Zm00001d004812](http://plants.ensembl.org/zea_mays/Gene/Summary?db=core;g=Zm00001d004812) | Calcium-dependent protein kinase ZmCPK11 | [2](http://plants.ensembl.org/zea_mays/contigview?chr=2) | [140809252](http://plants.ensembl.org/zea_mays/contigview?chr=2&vc_start=140809252&vc_end=140813969) | [140813969](http://plants.ensembl.org/zea_mays/contigview?chr=2&vc_start=140809252&vc_end=140813969) | - |
| EH_Opt | [Zm00001d011666](http://plants.ensembl.org/zea_mays/Gene/Summary?db=core;g=Zm00001d011666) | Putative calcium-dependent protein kinase family protein | [8](http://plants.ensembl.org/zea_mays/contigview?chr=8) | [158082120](http://plants.ensembl.org/zea_mays/contigview?chr=8&vc_start=158082120&vc_end=158084527) | [158084527](http://plants.ensembl.org/zea_mays/contigview?chr=8&vc_start=158082120&vc_end=158084527) | - |
| EH_Opt | [Zm00001d011673](http://plants.ensembl.org/zea_mays/Gene/Summary?db=core;g=Zm00001d011673) | Farnesyl pyrophosphate synthetase | [8](http://plants.ensembl.org/zea_mays/contigview?chr=8) | [158269867](http://plants.ensembl.org/zea_mays/contigview?chr=8&vc_start=158269867&vc_end=158274000) | [158274000](http://plants.ensembl.org/zea_mays/contigview?chr=8&vc_start=158269867&vc_end=158274000) | - |
| EH_LowN | [Zm00001d027885](http://plants.ensembl.org/zea_mays/Gene/Summary?db=core;g=Zm00001d027885) | YGGT family protein | [1](http://plants.ensembl.org/zea_mays/contigview?chr=1) | [16721226](http://plants.ensembl.org/zea_mays/contigview?chr=1&vc_start=16721226&vc_end=16722692) | [16722692](http://plants.ensembl.org/zea_mays/contigview?chr=1&vc_start=16721226&vc_end=16722692) | - |
| EH_LowN | [Zm00001d006061](http://plants.ensembl.org/zea_mays/Gene/Summary?db=core;g=Zm00001d006061) | NADH dehydrogenase [ubiquinone] 1 alpha subcomplex subunit 12 | [2](http://plants.ensembl.org/zea_mays/contigview?chr=2) | [196886906](http://plants.ensembl.org/zea_mays/contigview?chr=2&vc_start=196886906&vc_end=196890424) | [196890424](http://plants.ensembl.org/zea_mays/contigview?chr=2&vc_start=196886906&vc_end=196890424) | - |
| EH_LowN | [Zm00001d044054](http://plants.ensembl.org/zea_mays/Gene/Summary?db=core;g=Zm00001d044054) | GTP-binding nuclear protein | [3](http://plants.ensembl.org/zea_mays/contigview?chr=3) | [217928184](http://plants.ensembl.org/zea_mays/contigview?chr=3&vc_start=217928184&vc_end=217932236) | [217932236](http://plants.ensembl.org/zea_mays/contigview?chr=3&vc_start=217928184&vc_end=217932236) | - |
| EH_LowN | [Zm00001d044045](http://plants.ensembl.org/zea_mays/Gene/Summary?db=core;g=Zm00001d044045) | Esterase | [3](http://plants.ensembl.org/zea_mays/contigview?chr=3) | [217679351](http://plants.ensembl.org/zea_mays/contigview?chr=3&vc_start=217679351&vc_end=217682059) | [217682059](http://plants.ensembl.org/zea_mays/contigview?chr=3&vc_start=217679351&vc_end=217682059) | - |
| EH_LowN | [Zm00001d015292](http://plants.ensembl.org/zea_mays/Gene/Summary?db=core;g=Zm00001d015292) | Cellulase | [5](http://plants.ensembl.org/zea_mays/contigview?chr=5) | [82970088](http://plants.ensembl.org/zea_mays/contigview?chr=5&vc_start=82970088&vc_end=82974239) | [82974239](http://plants.ensembl.org/zea_mays/contigview?chr=5&vc_start=82970088&vc_end=82974239) | - |
| EH_LowN | [Zm00001d015300](http://plants.ensembl.org/zea_mays/Gene/Summary?db=core;g=Zm00001d015300) | Ribosomal protein L19 | [5](http://plants.ensembl.org/zea_mays/contigview?chr=5) | [83323891](http://plants.ensembl.org/zea_mays/contigview?chr=5&vc_start=83323891&vc_end=83326591) | [83326591](http://plants.ensembl.org/zea_mays/contigview?chr=5&vc_start=83323891&vc_end=83326591) | - |
| EH_LowN | [Zm00001d035143](http://plants.ensembl.org/zea_mays/Gene/Summary?db=core;g=Zm00001d035143) | Non-specific serine/threonine protein kinase | [6](http://plants.ensembl.org/zea_mays/contigview?chr=6) | [6884406](http://plants.ensembl.org/zea_mays/contigview?chr=6&vc_start=6884406&vc_end=6891194) | [6891194](http://plants.ensembl.org/zea_mays/contigview?chr=6&vc_start=6884406&vc_end=6891194) | - |
| EH_LowN | [Zm00001d025699](http://plants.ensembl.org/zea_mays/Gene/Summary?db=core;g=Zm00001d025699) | Nucleotide binding protein | [10](http://plants.ensembl.org/zea_mays/contigview?chr=10) | [126708804](http://plants.ensembl.org/zea_mays/contigview?chr=10&vc_start=126708804&vc_end=126710162) | [126710162](http://plants.ensembl.org/zea_mays/contigview?chr=10&vc_start=126708804&vc_end=126710162) | - |
| EH_LowN | [Zm00001d025704](http://plants.ensembl.org/zea_mays/Gene/Summary?db=core;g=Zm00001d025704) | Wax synthase isoform 1 | [10](http://plants.ensembl.org/zea_mays/contigview?chr=10) | [126794136](http://plants.ensembl.org/zea_mays/contigview?chr=10&vc_start=126794136&vc_end=126795161) | [126795161](http://plants.ensembl.org/zea_mays/contigview?chr=10&vc_start=126794136&vc_end=126795161) | - |
| EH_LowN | [Zm00001d026391](http://plants.ensembl.org/zea_mays/Gene/Summary?db=core;g=Zm00001d026391) | GNAT transcription factor | [10](http://plants.ensembl.org/zea_mays/contigview?chr=10) | [144991865](http://plants.ensembl.org/zea_mays/contigview?chr=10&vc_start=144991865&vc_end=144994560) | [144994560](http://plants.ensembl.org/zea_mays/contigview?chr=10&vc_start=144991865&vc_end=144994560) | - |
| EH_LowN | [Zm00001d026394](http://plants.ensembl.org/zea_mays/Gene/Summary?db=core;g=Zm00001d026394) | Hexosyltransferase | [10](http://plants.ensembl.org/zea_mays/contigview?chr=10) | [145133662](http://plants.ensembl.org/zea_mays/contigview?chr=10&vc_start=145133662&vc_end=145136374) | [145136374](http://plants.ensembl.org/zea_mays/contigview?chr=10&vc_start=145133662&vc_end=145136374) | - |
| EH_LowN | [Zm00001d026397](http://plants.ensembl.org/zea_mays/Gene/Summary?db=core;g=Zm00001d026397) | RNA-binding protein AKIP1 | [10](http://plants.ensembl.org/zea_mays/contigview?chr=10) | [145157330](http://plants.ensembl.org/zea_mays/contigview?chr=10&vc_start=145157330&vc_end=145158895) | [145158895](http://plants.ensembl.org/zea_mays/contigview?chr=10&vc_start=145157330&vc_end=145158895) | - |
| PH_Opt | [Zm00001d049822](http://plants.ensembl.org/zea_mays/Gene/Summary?db=core;g=Zm00001d049822) | Teosinte glume architecture 1 | [4](http://plants.ensembl.org/zea_mays/contigview?chr=4) | [46350597](http://plants.ensembl.org/zea_mays/contigview?chr=4&vc_start=46350597&vc_end=46355118) | [46355118](http://plants.ensembl.org/zea_mays/contigview?chr=4&vc_start=46350597&vc_end=46355118) | [TGA1](http://plants.ensembl.org/zea_mays/Gene/Summary?db=core;g=Zm00001d049822) |
| PH_Opt | [Zm00001d052254](http://plants.ensembl.org/zea_mays/Gene/Summary?db=core;g=Zm00001d052254) | DNA binding protein | [4](http://plants.ensembl.org/zea_mays/contigview?chr=4) | [184820400](http://plants.ensembl.org/zea_mays/contigview?chr=4&vc_start=184820400&vc_end=184821661) | [184821661](http://plants.ensembl.org/zea_mays/contigview?chr=4&vc_start=184820400&vc_end=184821661) | - |
| PH_Opt | [Zm00001d052260](http://plants.ensembl.org/zea_mays/Gene/Summary?db=core;g=Zm00001d052260) | Hexosyltransferase | [4](http://plants.ensembl.org/zea_mays/contigview?chr=4) | [185139277](http://plants.ensembl.org/zea_mays/contigview?chr=4&vc_start=185139277&vc_end=185141193) | [185141193](http://plants.ensembl.org/zea_mays/contigview?chr=4&vc_start=185139277&vc_end=185141193) | - |
| PH_Opt | [Zm00001d018819](http://plants.ensembl.org/zea_mays/Gene/Summary?db=core;g=Zm00001d018819) | Viviparous-14 | [7](http://plants.ensembl.org/zea_mays/contigview?chr=7) | [6342456](http://plants.ensembl.org/zea_mays/contigview?chr=7&vc_start=6342456&vc_end=6344361) | [6344361](http://plants.ensembl.org/zea_mays/contigview?chr=7&vc_start=6342456&vc_end=6344361) | - |
| PH_Opt | [Zm00001d026321](http://plants.ensembl.org/zea_mays/Gene/Summary?db=core;g=Zm00001d026321) | Kelch motif family protein | [10](http://plants.ensembl.org/zea_mays/contigview?chr=10) | [143570399](http://plants.ensembl.org/zea_mays/contigview?chr=10&vc_start=143570399&vc_end=143571745) | [143571745](http://plants.ensembl.org/zea_mays/contigview?chr=10&vc_start=143570399&vc_end=143571745) | - |
| PH_LowN | [Zm00001d027919](http://plants.ensembl.org/zea_mays/Gene/Summary?db=core;g=Zm00001d027919) | 40S ribosomal protein SA | [1](http://plants.ensembl.org/zea_mays/contigview?chr=1) | [17615562](http://plants.ensembl.org/zea_mays/contigview?chr=1&vc_start=17615562&vc_end=17626036) | [17626036](http://plants.ensembl.org/zea_mays/contigview?chr=1&vc_start=17615562&vc_end=17626036) | - |
| PH_LowN | [Zm00001d027921](http://plants.ensembl.org/zea_mays/Gene/Summary?db=core;g=Zm00001d027921) | Protein DETOXIFICATION | [1](http://plants.ensembl.org/zea_mays/contigview?chr=1) | [17697601](http://plants.ensembl.org/zea_mays/contigview?chr=1&vc_start=17697601&vc_end=17709046) | [17709046](http://plants.ensembl.org/zea_mays/contigview?chr=1&vc_start=17697601&vc_end=17709046) | - |
| PH_LowN | [Zm00001d027922](http://plants.ensembl.org/zea_mays/Gene/Summary?db=core;g=Zm00001d027922) | Eukaryotic translation initiation factor 3 subunit K | [1](http://plants.ensembl.org/zea_mays/contigview?chr=1) | [17722776](http://plants.ensembl.org/zea_mays/contigview?chr=1&vc_start=17722776&vc_end=17726155) | [17726155](http://plants.ensembl.org/zea_mays/contigview?chr=1&vc_start=17722776&vc_end=17726155) | - |
| PH_LowN | [Zm00001d027924](http://plants.ensembl.org/zea_mays/Gene/Summary?db=core;g=Zm00001d027924) | AP2-EREBP transcription factor | [1](http://plants.ensembl.org/zea_mays/contigview?chr=1) | [17775779](http://plants.ensembl.org/zea_mays/contigview?chr=1&vc_start=17775779&vc_end=17777344) | [17777344](http://plants.ensembl.org/zea_mays/contigview?chr=1&vc_start=17775779&vc_end=17777344) | - |
| PH_LowN | [Zm00001d043422](http://plants.ensembl.org/zea_mays/Gene/Summary?db=core;g=Zm00001d043422) | RING-H2 finger protein ATL2K | [3](http://plants.ensembl.org/zea_mays/contigview?chr=3) | [199163496](http://plants.ensembl.org/zea_mays/contigview?chr=3&vc_start=199163496&vc_end=199164221) | [199164221](http://plants.ensembl.org/zea_mays/contigview?chr=3&vc_start=199163496&vc_end=199164221) | - |
| PH_LowN | [Zm00001d043420](http://plants.ensembl.org/zea_mays/Gene/Summary?db=core;g=Zm00001d043420) | BZIP transcription factor | [3](http://plants.ensembl.org/zea_mays/contigview?chr=3) | [199110559](http://plants.ensembl.org/zea_mays/contigview?chr=3&vc_start=199110559&vc_end=199113757) | [199113757](http://plants.ensembl.org/zea_mays/contigview?chr=3&vc_start=199110559&vc_end=199113757) | - |
| PH_LowN | [Zm00001d053632](http://plants.ensembl.org/zea_mays/Gene/Summary?db=core;g=Zm00001d053632) | 40S ribosomal protein S8 | [4](http://plants.ensembl.org/zea_mays/contigview?chr=4) | [237551123](http://plants.ensembl.org/zea_mays/contigview?chr=4&vc_start=237551123&vc_end=237553636) | [237553636](http://plants.ensembl.org/zea_mays/contigview?chr=4&vc_start=237551123&vc_end=237553636) | [rps8](http://plants.ensembl.org/zea_mays/Gene/Summary?db=core;g=Zm00001d053632) |
| PH_LowN | [Zm00001d008914](http://plants.ensembl.org/zea_mays/Gene/Summary?db=core;g=Zm00001d008914) | Putative RING zinc finger domain superfamily protein | [8](http://plants.ensembl.org/zea_mays/contigview?chr=8) | [25177981](http://plants.ensembl.org/zea_mays/contigview?chr=8&vc_start=25177981&vc_end=25178538) | [25178538](http://plants.ensembl.org/zea_mays/contigview?chr=8&vc_start=25177981&vc_end=25178538) | - |
| PH_LowN | [Zm00001d008916](http://plants.ensembl.org/zea_mays/Gene/Summary?db=core;g=Zm00001d008916) | Small nuclear ribonucleoprotein E | [8](http://plants.ensembl.org/zea_mays/contigview?chr=8) | [25186392](http://plants.ensembl.org/zea_mays/contigview?chr=8&vc_start=25186392&vc_end=25205767) | [25205767](http://plants.ensembl.org/zea_mays/contigview?chr=8&vc_start=25186392&vc_end=25205767) | - |
| PH_LowN | [Zm00001d008918](http://plants.ensembl.org/zea_mays/Gene/Summary?db=core;g=Zm00001d008918) | SURF1-like protein | [8](http://plants.ensembl.org/zea_mays/contigview?chr=8) | [25221056](http://plants.ensembl.org/zea_mays/contigview?chr=8&vc_start=25221056&vc_end=25222003) | [25222003](http://plants.ensembl.org/zea_mays/contigview?chr=8&vc_start=25221056&vc_end=25222003) | - |
| EPO_Opt | [Zm00001d029448](http://plants.ensembl.org/zea_mays/Gene/Summary?db=core;g=Zm00001d029448) | TIFY6 | [1](http://plants.ensembl.org/zea_mays/contigview?chr=1) | [71161670](http://plants.ensembl.org/zea_mays/contigview?chr=1&vc_start=71161670&vc_end=71164215) | [71164215](http://plants.ensembl.org/zea_mays/contigview?chr=1&vc_start=71161670&vc_end=71164215) | - |
| EPO_Opt | [Zm00001d009589](http://plants.ensembl.org/zea_mays/Gene/Summary?db=core;g=Zm00001d009589) | Chlorophyll a-b binding protein, chloroplastic | [8](http://plants.ensembl.org/zea_mays/contigview?chr=8) | [71899226](http://plants.ensembl.org/zea_mays/contigview?chr=8&vc_start=71899226&vc_end=71900023) | [71900023](http://plants.ensembl.org/zea_mays/contigview?chr=8&vc_start=71899226&vc_end=71900023) | - |
| EPO_Opt | [Zm00001d009595](http://plants.ensembl.org/zea_mays/Gene/Summary?db=core;g=Zm00001d009595) | Putative WRKY DNA-binding domain superfamily protein | [8](http://plants.ensembl.org/zea_mays/contigview?chr=8) | [72169414](http://plants.ensembl.org/zea_mays/contigview?chr=8&vc_start=72169414&vc_end=72173597) | [72173597](http://plants.ensembl.org/zea_mays/contigview?chr=8&vc_start=72169414&vc_end=72173597) | - |
| EPO_Opt | [Zm00001d026326](http://plants.ensembl.org/zea_mays/Gene/Summary?db=core;g=Zm00001d026326) | F-box domain containing protein | [10](http://plants.ensembl.org/zea_mays/contigview?chr=10) | [143599140](http://plants.ensembl.org/zea_mays/contigview?chr=10&vc_start=143599140&vc_end=143600834) | [143600834](http://plants.ensembl.org/zea_mays/contigview?chr=10&vc_start=143599140&vc_end=143600834) | - |
| EPO_Opt | [Zm00001d026335](http://plants.ensembl.org/zea_mays/Gene/Summary?db=core;g=Zm00001d026335) | Autophagy-related protein | [10](http://plants.ensembl.org/zea_mays/contigview?chr=10) | [143747463](http://plants.ensembl.org/zea_mays/contigview?chr=10&vc_start=143747463&vc_end=143750718) | [143750718](http://plants.ensembl.org/zea_mays/contigview?chr=10&vc_start=143747463&vc_end=143750718) | - |
| EPO_Opt | [Zm00001d026337](http://plants.ensembl.org/zea_mays/Gene/Summary?db=core;g=Zm00001d026337) | Starch synthase IIIb-1 | [10](http://plants.ensembl.org/zea_mays/contigview?chr=10) | [143786832](http://plants.ensembl.org/zea_mays/contigview?chr=10&vc_start=143786832&vc_end=143796162) | [143796162](http://plants.ensembl.org/zea_mays/contigview?chr=10&vc_start=143786832&vc_end=143796162) | - |
| EPO_LowN | [Zm00001d031933](http://plants.ensembl.org/zea_mays/Gene/Summary?db=core;g=Zm00001d031933) | Signal peptide peptidase-like 3 | [1](http://plants.ensembl.org/zea_mays/contigview?chr=1) | [207276632](http://plants.ensembl.org/zea_mays/contigview?chr=1&vc_start=207276632&vc_end=207281619) | [207281619](http://plants.ensembl.org/zea_mays/contigview?chr=1&vc_start=207276632&vc_end=207281619) | - |
| EPO_LowN | [Zm00001d034143](http://plants.ensembl.org/zea_mays/Gene/Summary?db=core;g=Zm00001d034143) | GNAT transcription factor | [1](http://plants.ensembl.org/zea_mays/contigview?chr=1) | [285008208](http://plants.ensembl.org/zea_mays/contigview?chr=1&vc_start=285008208&vc_end=285010916) | [285010916](http://plants.ensembl.org/zea_mays/contigview?chr=1&vc_start=285008208&vc_end=285010916) | - |
| EPO_LowN | [Zm00001d034160](http://plants.ensembl.org/zea_mays/Gene/Summary?db=core;g=Zm00001d034160) | DNA binding protein | [1](http://plants.ensembl.org/zea_mays/contigview?chr=1) | [285335178](http://plants.ensembl.org/zea_mays/contigview?chr=1&vc_start=285335178&vc_end=285337132) | [285337132](http://plants.ensembl.org/zea_mays/contigview?chr=1&vc_start=285335178&vc_end=285337132) | - |
| EPO_LowN | [Zm00001d036698](http://plants.ensembl.org/zea_mays/Gene/Summary?db=core;g=Zm00001d036698) | Chloroplast pentatricopeptide repeat protein 10 | [6](http://plants.ensembl.org/zea_mays/contigview?chr=6) | [98069169](http://plants.ensembl.org/zea_mays/contigview?chr=6&vc_start=98069169&vc_end=98071529) | [98071529](http://plants.ensembl.org/zea_mays/contigview?chr=6&vc_start=98069169&vc_end=98071529) | - |
| EPO_LowN | [Zm00001d036700](http://plants.ensembl.org/zea_mays/Gene/Summary?db=core;g=Zm00001d036700) | CASP-like protein | [6](http://plants.ensembl.org/zea_mays/contigview?chr=6) | [98074454](http://plants.ensembl.org/zea_mays/contigview?chr=6&vc_start=98074454&vc_end=98078079) | [98078079](http://plants.ensembl.org/zea_mays/contigview?chr=6&vc_start=98074454&vc_end=98078079) | - |
| EPO_LowN | [Zm00001d025614](http://plants.ensembl.org/zea_mays/Gene/Summary?db=core;g=Zm00001d025614) | Putative IQ calmodulin-binding and BAG domain containing family protein | [10](http://plants.ensembl.org/zea_mays/contigview?chr=10) | [123911243](http://plants.ensembl.org/zea_mays/contigview?chr=10&vc_start=123911243&vc_end=123912298) | [123912298](http://plants.ensembl.org/zea_mays/contigview?chr=10&vc_start=123911243&vc_end=123912298) | - |
| EPO_LowN | [Zm00001d025616](http://plants.ensembl.org/zea_mays/Gene/Summary?db=core;g=Zm00001d025616) | Plastid-specific 30S ribosomal protein 3 | [10](http://plants.ensembl.org/zea_mays/contigview?chr=10) | [124037257](http://plants.ensembl.org/zea_mays/contigview?chr=10&vc_start=124037257&vc_end=124039095) | [124039095](http://plants.ensembl.org/zea_mays/contigview?chr=10&vc_start=124037257&vc_end=124039095) | - |
| EPP_LowN | [Zm00001d051891](http://plants.ensembl.org/zea_mays/Gene/Summary?db=core;g=Zm00001d051891) | Putative LOB domain-containing family protein | [4](http://plants.ensembl.org/zea_mays/contigview?chr=4) | [173818577](http://plants.ensembl.org/zea_mays/contigview?chr=4&vc_start=173818577&vc_end=173819660) | [173819660](http://plants.ensembl.org/zea_mays/contigview?chr=4&vc_start=173818577&vc_end=173819660) | - |
| EPP_LowN | [Zm00001d051892](http://plants.ensembl.org/zea_mays/Gene/Summary?db=core;g=Zm00001d051892) | Anthocyanin 5-aromatic acyltransferase | [4](http://plants.ensembl.org/zea_mays/contigview?chr=4) | [173824341](http://plants.ensembl.org/zea_mays/contigview?chr=4&vc_start=173824341&vc_end=173825720) | [173825720](http://plants.ensembl.org/zea_mays/contigview?chr=4&vc_start=173824341&vc_end=173825720) | - |
| EPP_LowN | [Zm00001d051894](http://plants.ensembl.org/zea_mays/Gene/Summary?db=core;g=Zm00001d051894) | Putative RING zinc finger domain superfamily protein | [4](http://plants.ensembl.org/zea_mays/contigview?chr=4) | [173865361](http://plants.ensembl.org/zea_mays/contigview?chr=4&vc_start=173865361&vc_end=173866293) | [173866293](http://plants.ensembl.org/zea_mays/contigview?chr=4&vc_start=173865361&vc_end=173866293) | - |
| EPP_LowN | [Zm00001d051898](http://plants.ensembl.org/zea_mays/Gene/Summary?db=core;g=Zm00001d051898) | Serine/threonine-protein phosphatase | [4](http://plants.ensembl.org/zea_mays/contigview?chr=4) | [173925846](http://plants.ensembl.org/zea_mays/contigview?chr=4&vc_start=173925846&vc_end=173928349) | [173928349](http://plants.ensembl.org/zea_mays/contigview?chr=4&vc_start=173925846&vc_end=173928349) | - |
| EPP_LowN | [Zm00001d017199](http://plants.ensembl.org/zea_mays/Gene/Summary?db=core;g=Zm00001d017199) | Temperature-induced lipocalin-1 | [5](http://plants.ensembl.org/zea_mays/contigview?chr=5) | [188679379](http://plants.ensembl.org/zea_mays/contigview?chr=5&vc_start=188679379&vc_end=188680432) | [188680432](http://plants.ensembl.org/zea_mays/contigview?chr=5&vc_start=188679379&vc_end=188680432) | - |
| EPP_LowN | [Zm00001d026569](http://plants.ensembl.org/zea_mays/Gene/Summary?db=core;g=Zm00001d026569) | Acyl-[acyl-carrier-protein] hydrolase | [10](http://plants.ensembl.org/zea_mays/contigview?chr=10) | [148186747](http://plants.ensembl.org/zea_mays/contigview?chr=10&vc_start=148186747&vc_end=148188802) | [148188802](http://plants.ensembl.org/zea_mays/contigview?chr=10&vc_start=148186747&vc_end=148188802) | - |
| EPP_LowN | [Zm00001d026575](http://plants.ensembl.org/zea_mays/Gene/Summary?db=core;g=Zm00001d026575) | Reticulon-like protein | [10](http://plants.ensembl.org/zea_mays/contigview?chr=10) | [148249658](http://plants.ensembl.org/zea_mays/contigview?chr=10&vc_start=148249658&vc_end=148253209) | [148253209](http://plants.ensembl.org/zea_mays/contigview?chr=10&vc_start=148249658&vc_end=148253209) | - |
| EPP_LowN | [Zm00001d026576](http://plants.ensembl.org/zea_mays/Gene/Summary?db=core;g=Zm00001d026576) | Leucine-rich repeat (LRR) family protein | [10](http://plants.ensembl.org/zea_mays/contigview?chr=10) | [148260149](http://plants.ensembl.org/zea_mays/contigview?chr=10&vc_start=148260149&vc_end=148261444) | [148261444](http://plants.ensembl.org/zea_mays/contigview?chr=10&vc_start=148260149&vc_end=148261444) | - |
| EPP_LowN | [Zm00001d026577](http://plants.ensembl.org/zea_mays/Gene/Summary?db=core;g=Zm00001d026577) | Cysteine protease 1 | [10](http://plants.ensembl.org/zea_mays/contigview?chr=10) | [148261491](http://plants.ensembl.org/zea_mays/contigview?chr=10&vc_start=148261491&vc_end=148265297) | [148265297](http://plants.ensembl.org/zea_mays/contigview?chr=10&vc_start=148261491&vc_end=148265297) | - |
| EPP_LowN | [Zm00001d026578](http://plants.ensembl.org/zea_mays/Gene/Summary?db=core;g=Zm00001d026578) | 60S acidic ribosomal protein P2A | [10](http://plants.ensembl.org/zea_mays/contigview?chr=10) | [148267758](http://plants.ensembl.org/zea_mays/contigview?chr=10&vc_start=148267758&vc_end=148269932) | [148269932](http://plants.ensembl.org/zea_mays/contigview?chr=10&vc_start=148267758&vc_end=148269932) | - |
| EPP_LowN | [Zm00001d026587](http://plants.ensembl.org/zea_mays/Gene/Summary?db=core;g=Zm00001d026587) | Zinc finger C-x8-C-x5-C-x3-H type family protein | [10](http://plants.ensembl.org/zea_mays/contigview?chr=10) | [148406795](http://plants.ensembl.org/zea_mays/contigview?chr=10&vc_start=148406795&vc_end=148411190) | [148411190](http://plants.ensembl.org/zea_mays/contigview?chr=10&vc_start=148406795&vc_end=148411190) | - |
| SEN_Opt | [Zm00001d052034](http://plants.ensembl.org/zea_mays/Gene/Summary?db=core;g=Zm00001d052034) | Thioredoxin H-type 5 | [4](http://plants.ensembl.org/zea_mays/contigview?chr=4) | [177028520](http://plants.ensembl.org/zea_mays/contigview?chr=4&vc_start=177028520&vc_end=177029881) | [177029881](http://plants.ensembl.org/zea_mays/contigview?chr=4&vc_start=177028520&vc_end=177029881) | - |
| SEN_Opt | [Zm00001d052040](http://plants.ensembl.org/zea_mays/Gene/Summary?db=core;g=Zm00001d052040) | Cytochrome c oxidase copper chaperone | [4](http://plants.ensembl.org/zea_mays/contigview?chr=4) | [177078013](http://plants.ensembl.org/zea_mays/contigview?chr=4&vc_start=177078013&vc_end=177079956) | [177079956](http://plants.ensembl.org/zea_mays/contigview?chr=4&vc_start=177078013&vc_end=177079956) | - |
| SEN_Opt | [Zm00001d052043](http://plants.ensembl.org/zea_mays/Gene/Summary?db=core;g=Zm00001d052043) | Auxin-responsive protein | [4](http://plants.ensembl.org/zea_mays/contigview?chr=4) | [177090545](http://plants.ensembl.org/zea_mays/contigview?chr=4&vc_start=177090545&vc_end=177102253) | [177102253](http://plants.ensembl.org/zea_mays/contigview?chr=4&vc_start=177090545&vc_end=177102253) | - |
| SEN_Opt | [Zm00001d052044](http://plants.ensembl.org/zea_mays/Gene/Summary?db=core;g=Zm00001d052044) | Putative RING zinc finger domain superfamily protein | [4](http://plants.ensembl.org/zea_mays/contigview?chr=4) | [177336257](http://plants.ensembl.org/zea_mays/contigview?chr=4&vc_start=177336257&vc_end=177340312) | [177340312](http://plants.ensembl.org/zea_mays/contigview?chr=4&vc_start=177336257&vc_end=177340312) | - |
| SEN_Opt | [Zm00001d013306](http://plants.ensembl.org/zea_mays/Gene/Summary?db=core;g=Zm00001d013306) | PRA1 family protein | [5](http://plants.ensembl.org/zea_mays/contigview?chr=5) | [8172127](http://plants.ensembl.org/zea_mays/contigview?chr=5&vc_start=8172127&vc_end=8172948) | [8172948](http://plants.ensembl.org/zea_mays/contigview?chr=5&vc_start=8172127&vc_end=8172948) | - |
| SEN_Opt | [Zm00001d013307](http://plants.ensembl.org/zea_mays/Gene/Summary?db=core;g=Zm00001d013307) | WRKY transcription factor | [5](http://plants.ensembl.org/zea_mays/contigview?chr=5) | [8180118](http://plants.ensembl.org/zea_mays/contigview?chr=5&vc_start=8180118&vc_end=8184577) | [8184577](http://plants.ensembl.org/zea_mays/contigview?chr=5&vc_start=8180118&vc_end=8184577) | - |
| SEN_Opt | [Zm00001d013309](http://plants.ensembl.org/zea_mays/Gene/Summary?db=core;g=Zm00001d013309) | Ribosomal protein S10 | [5](http://plants.ensembl.org/zea_mays/contigview?chr=5) | [8293227](http://plants.ensembl.org/zea_mays/contigview?chr=5&vc_start=8293227&vc_end=8293556) | [8293556](http://plants.ensembl.org/zea_mays/contigview?chr=5&vc_start=8293227&vc_end=8293556) | - |
| SEN_Opt | [Zm00001d011708](http://plants.ensembl.org/zea_mays/Gene/Summary?db=core;g=Zm00001d011708) | Putative uncharacterized protein | [8](http://plants.ensembl.org/zea_mays/contigview?chr=8) | [159401583](http://plants.ensembl.org/zea_mays/contigview?chr=8&vc_start=159401583&vc_end=159404367) | [159404367](http://plants.ensembl.org/zea_mays/contigview?chr=8&vc_start=159401583&vc_end=159404367) | - |
| SEN_Opt | [Zm00001d011710](http://plants.ensembl.org/zea_mays/Gene/Summary?db=core;g=Zm00001d011710) | Cytidine deaminase | [8](http://plants.ensembl.org/zea_mays/contigview?chr=8) | [159460166](http://plants.ensembl.org/zea_mays/contigview?chr=8&vc_start=159460166&vc_end=159461104) | [159461104](http://plants.ensembl.org/zea_mays/contigview?chr=8&vc_start=159460166&vc_end=159461104) | - |
| SEN_Opt | [Zm00001d011721](http://plants.ensembl.org/zea_mays/Gene/Summary?db=core;g=Zm00001d011721) | Putative leucine-rich repeat receptor-like protein kinase family protein | [8](http://plants.ensembl.org/zea_mays/contigview?chr=8) | [159899428](http://plants.ensembl.org/zea_mays/contigview?chr=8&vc_start=159899428&vc_end=159902796) | [159902796](http://plants.ensembl.org/zea_mays/contigview?chr=8&vc_start=159899428&vc_end=159902796) | - |
| SEN_Opt | [Zm00001d048252](http://plants.ensembl.org/zea_mays/Gene/Summary?db=core;g=Zm00001d048252) | Eukaryotic translation initiation factor 3 subunit K | [9](http://plants.ensembl.org/zea_mays/contigview?chr=9) | [153250495](http://plants.ensembl.org/zea_mays/contigview?chr=9&vc_start=153250495&vc_end=153253737) | [153253737](http://plants.ensembl.org/zea_mays/contigview?chr=9&vc_start=153250495&vc_end=153253737) | - |
| SEN_Opt | [Zm00001d048253](http://plants.ensembl.org/zea_mays/Gene/Summary?db=core;g=Zm00001d048253) | 40S ribosomal protein SA | [9](http://plants.ensembl.org/zea_mays/contigview?chr=9) | [153255360](http://plants.ensembl.org/zea_mays/contigview?chr=9&vc_start=153255360&vc_end=153257954) | [153257954](http://plants.ensembl.org/zea_mays/contigview?chr=9&vc_start=153255360&vc_end=153257954) | - |
| SEN_Opt | [Zm00001d048268](http://plants.ensembl.org/zea_mays/Gene/Summary?db=core;g=Zm00001d048268) | Putative tify domain/CCT motif transcription factor family protein | [9](http://plants.ensembl.org/zea_mays/contigview?chr=9) | [153485703](http://plants.ensembl.org/zea_mays/contigview?chr=9&vc_start=153485703&vc_end=153486254) | [153486254](http://plants.ensembl.org/zea_mays/contigview?chr=9&vc_start=153485703&vc_end=153486254) | - |
| SEN_Opt | [Zm00001d048271](http://plants.ensembl.org/zea_mays/Gene/Summary?db=core;g=Zm00001d048271) | Proteasome subunit alpha type | [9](http://plants.ensembl.org/zea_mays/contigview?chr=9) | [153586355](http://plants.ensembl.org/zea_mays/contigview?chr=9&vc_start=153586355&vc_end=153591283) | [153591283](http://plants.ensembl.org/zea_mays/contigview?chr=9&vc_start=153586355&vc_end=153591283) | - |
